# Immediate vs Gradual Brace Weaning Protocols in Adolescent Idiopathic Scoliosis: A Randomized Clinical Trial

**DOI:** 10.1001/jamapediatrics.2024.1484

**Published:** 2024-06-03

**Authors:** Prudence Wing Hang Cheung, Oi Kiu Olivia Chan, Hao Wu, Marcus Kin Long Lai, Lester Po Kwan Wong, Shiyu Tang, Jason Pui Yin Cheung

**Affiliations:** 1 Department of Orthopaedics and Traumatology, The University of Hong Kong, Pokfulam, Hong Kong SAR, China

## Abstract

**Importance:**

Lack of evidence and consensus for brace weaning protocol in adolescent idiopathic scoliosis (AIS) results in clinicians prescribing gradual weaning in the hope of avoiding curve deterioration after weaning. However, gradual weaning contributes to prolonged brace wear, which can affect spinal stiffness and health-related quality of life (HRQoL).

**Objective:**

To determine whether gradual weaning results in better curve magnitude and truncal balance maintenance after brace weaning vs immediate brace removal for patients with AIS.

**Design, Setting, and Participants:**

This was an open-labeled randomized clinical trial commenced in April 2017 with 24-month follow-up completed in January 2023. Outcome assessors were masked to weaning protocol assigned. The study took place at a territory-wide tertiary scoliosis clinic serving the largest number of referrals in the local population. Patients with AIS ready to wean off of brace wear were eligible (402 were screened; 33 were excluded [15 for <18 hours/day of brace-wear compliance before weaning, 11 were treated with Milwaukee brace, and 7 declined to participate]; and 369 were included), and those who were treated with a custom molded thoracolumbosacral orthosis and had reached skeletal maturity were consecutively recruited.

**Interventions:**

Patients were randomized to gradual weaning protocol (n = 176) with an additional 6 months of nighttime wear before completely stopping or immediate weaning protocol (n = 193) with immediate brace removal at recruitment.

**Main Outcomes and Measures:**

Changes in major curve Cobb angle and truncal balance from the time of weaning to 6-month, 12-month, and 24-month follow-up. HRQoL was also assessed using the refined Scoliosis Research Society 22-item and EuroQol 5-dimension questionnaires.

**Results:**

A total of 369 patients (mean [SD] age, 14.9 [1.1] years; 304 [83.4%] girls) were randomized with 284 (77.0%) completing 24-month longitudinal follow-up. Immediate and gradual weaning groups had no significant differences in change of major Cobb angle at postweaning 6-month (difference, −0.6°; 95% CI, −1.4 to 0.2; *P* = .17), 12-month (difference, −0.3°; 95% CI, −1.2 to 0.6; *P* = .47), and 24-month (difference, −0.3°; 95% CI, −1.2 to 0.7; *P* = .60) follow-up. The number of curve progression, nonprogression, and rebound cases were comparable (χ^^2^^_2_ = 2.123; *P* = .35). Postweaning changes in truncal balance and HRQoL demonstrated no significant differences between groups.

**Conclusions:**

Gradual weaning did not demonstrate superiority to immediate weaning with predefined criteria of Cobb angle and truncal balance maintenance and HRQoL after brace weaning. Gradual and immediate weaning achieved very similar maintenance of brace outcomes in AIS. We therefore recommend the consideration of immediate brace weaning, which aims to benefit patients with earlier time for increased exercises and activity level.

**Trial Registration:**

ClinicalTrials.gov Identifier: NCT03329716

## Key Points

**Question:**

Does gradual weaning of brace wear result in better curve magnitude maintenance than immediate weaning in adolescent idiopathic scoliosis (AIS)?

**Findings:**

In this randomized clinical trial of 369 patients with AIS, immediate and gradual weaning protocols had no significant differences in the change of major curve Cobb angle at 6, 12, and 24 months postweaning in the intention-to-treat population. Similar results were observed in the per-protocol analysis.

**Meaning:**

The findings suggest that immediate and gradual weaning can achieve similar maintenance of brace outcomes; hence immediate weaning can be considered, as it can benefit patients from earlier resumption of exercise and activity level.

## Teaser

This randomized clinical trial compares gradual weaning vs immediate brace removal in patients with adolescent idiopathic scoliosis.

## Introduction

For adolescent idiopathic scoliosis (AIS), bracing is the most common treatment modality for controlling curve progression with efficacy and effectiveness supported by high-level evidence.^1,2,3,4^ Its main purpose is to prevent curves from reaching the 50° surgical threshold^5^ and the 40° adulthood curve deterioration threshold at skeletal maturity.^5,6^ Brace treatment is completed at the end of skeletal growth as curve progression risk considerably decreases.^7,8^ Prolonged bracing can have drawbacks including reduced spinal mobility^9^ leading to poor body appearance and self-esteem with worsened health-related quality of life (HRQoL),^10,11,12^ muscle weakness,^13,14,15^ and osteoporosis.^16,17^

Despite having accurate parameters for determining when patients reach skeletal maturity and no longer require further bracing,^18^ there is a lack of consensus and evidence-based guidelines for how to wean brace wear. Brace weaning can be a slow process whereby daily brace-wear hours are gradually reduced or changed to night use only vs a rapid immediate brace removal. Some clinicians err on the side of gradual weaning to be certain that curves remain stable after some months of reduced brace hours,^19^ whereas others prefer immediate weaning for earlier return to normality with complete resumption of exercises, muscle training, and activity level.^20^ The main rationale for gradual weaning over a 6-month period is to allow spine adaptation to the unloaded environment while still maintaining the corrective posture. The benefit of gradual weaning is unclear, but immediate weaning may lead to worse Cobb angle and truncal imbalance and may be associated with worse HRQoL. This lack of evidence for an optimal brace weaning protocol is a serious limitation in current management of AIS, and it is imperative to deliver the best brace treatment protocol.

The primary aim of this trial was to compare the degree of Cobb angle maintenance between immediate and gradual brace weaning protocols for patients with AIS after brace weaning. The secondary aim was to compare the degree of truncal balance maintenance after weaning between immediate and gradual weaning protocols. We hypothesized that gradual weaning allows better adaptation of the spine to an unloaded environment, thereby resulting in better maintenance of Cobb angle and overall truncal balance even after brace removal. In addition, this trial aimed to determine any differences in HRQoL after weaning between immediate and gradual weaning protocols.

## Methods

### Trial Design and Patient Population

This was an open-labeled, randomized clinical trial conducted at the largest territory-wide tertiary referral scoliosis center for comparing 2 brace weaning protocols in AIS: immediate removal of brace and gradual brace weaning with nocturnal brace wear. The first patient was enrolled on April 24, 2017, and the last patient on September 28, 2020. The last postweaning 24-month follow-up was completed on January 30, 2023. Ethics approval from the institutional review board of the University of Hong Kong/Hospital Authority Hong Kong West Cluster and written informed consent conforming to the Declaration of Helsinki^21^ were obtained. This study followed the Consolidated Standards of Reporting Trials (CONSORT) reporting guideline.

Patients diagnosed with AIS who were ready to discontinue brace wear were eligible for this study. Inclusion criteria included patients with AIS who had been treated with a custom molded thoracolumbosacral orthosis and were ready for brace weaning as determined by having reached skeletal maturity based on the Scoliosis Research Society (SRS) criteria: Risser stage 4 or greater, more than 2 years postmenarche for girls, and no bodily growth between 2 visits. Patients must be previously referred for bracing according to SRS bracing criteria,^22^ with good brace-wear compliance throughout the course of brace treatment (≥18 hours/day). Patients who fulfilled study criteria and agreed to participate were consecutively recruited on the day of clinical decision of brace weaning. Follow-up consultations with radiographic examination were scheduled for every 6 months as per clinical routine. All included patients were monitored longitudinally to the primary end point at 12 months after brace weaning was issued. The final assessment was at 24 months to assess longer-term efficacy^23^ of the weaning protocol. The tudy protocol and eligibility criteria in full details are in Supplement 1.

### Randomization, Weaning Protocol, and Masking

Both patients and attending spine specialists were blinded from randomization sequence prior to consent. Block randomization with a randomly selected even number of block sizes was used. A master randomization list generated a priori by a biostatistician was managed by project coordinator, who concealed it to all researchers until study database closure. Outcome assessors were blinded to patient details and randomization sequence and were masked to weaning protocol assigned.

Patients were randomly allocated by project coordinator according to the master randomization list to either of the 2 groups: gradual brace weaning with brace-wear time shortened to night wearing for 6 additional months before completely stopping bracing or immediate brace weaning on the day of patient recruitment. For the gradual weaning group, brace-wear compliance (hours/day) was recorded by thermal sensors. Sufficient treatment compliance was defined as having completed brace wear for 8 or more hours per day for more than 80% of the 6-month gradual weaning period.

### Study Outcomes

The primary outcome was the change in major curve Cobb angle from the time of weaning (baseline) to 6-month, 12-month, and 24-month follow-up. Curve progression was examined as a greater-than 5° increase of major Cobb angle between follow-up time points and baseline. Secondary outcomes were the changes in truncal balance measured by truncal shift and listing (C7-central sacral vertebral line deviation) from baseline to 6-month, 12-month, and 24-month follow-up. Secondary outcomes also included the refined SRS 22-item (SRS-22r) questionnaire total score, EuroQol 5-dimension (EQ-5D) utility score, and EuroQol-visual analogue scale (EQ-VAS).

All study outcomes were assessed at baseline and at postweaning 6, 12, and 24 months. Skeletal maturity was routinely determined at weaning using Risser staging,^24^ distal radius and ulna (DRU) classification,^25^ and Sanders staging.^26^ In addition to Cobb angle and truncal balance, radiological parameters including T1 tilt, shoulder height, sagittal vertical axis, thoracic kyphosis, and lumbar lordosis were independently measured by 2 assessors. Interrater and intrarater reliabilities were satisfactory (intraclass correlation coefficient > 0.8).

### Statistical Analyses

According to Shi et al,^27^ 200 participants in their study cohort (46.5%) experienced curve progression at 2 years postweaning with a mean (SD) 5.1° (6.5°) increase of major Cobb angle. Based on this result with an equivalence margin of 0.4, a difference of 2° between the changes in Cobb angle of the 2 groups was considered clinically relevant. Assuming an SD of 6.5°, a sample size of 128 per group was required to detect an intergroup difference with 80% power at a .05 significance level. Accounting for 20% attrition, a total of 308 patients (154 per group) was proposed for recruitment. Due to concerns of follow-up rates during the COVID-19 pandemic, an increased recruitment by 20% was carried out despite the fact that the proposed sample size was fulfilled. Potential bias in relation to any discrepancy of the number of patients allocated to each trial arm was addressed by the analysis of patient profile at the time of weaning (eTable 1 in Supplement 2).

#### Primary Outcome

As larger Cobb angle at weaning relates to curve progression,^27,28^ major Cobb angles at weaning were first compared between groups and found comparable. Primary analysis using independent-samples *t* test determined the significance of differences in Cobb angle change between the 2 arms. The analysis was conducted according to intention-to-treat (ITT) and per-protocol (PP) principle^29^ (statistical plan in Supplement 1). PP analysis included all randomized patients with no major protocol deviations clinically significant to efficacy outcome, no treatment during follow-up, and sufficient treatment compliance for gradual weaning.

Secondary analyses included intergroup comparisons using 1-way analysis of covariance (ANCOVA) with post hoc Bonferroni correction to analyze the effect of weaning protocols on the outcomes of changes of major Cobb angles while adjusting for baseline covariates,^30^ which were identified via correlation tests (eTable 2 in Supplement 2). The moderately to strongly correlated DRU and Sanders stages were reduced into 1 component score (eTable 2 in Supplement 2) to avoid multicollinearity.

Subgroup analyses examined curve progression rate at postweaning 24 months in the PP population. Multivariable logistic regressions were used to examine whether weaning protocol (immediate vs gradual), skeletal maturity status at weaning, and any baseline parameters were associated with curve progression. The occurrence of progression, nonprogression or rebound (with >5° major Cobb angle increase at follow-up but major Cobb angle at weaning was less than at prebrace) was compared between protocols using the Pearson χ^2^ test.

#### Secondary Outcomes

Primary and secondary analyses (correlated covariates in eTable 3 in Supplement 2) were conducted to determine whether there was any significant difference of changes of truncal balance and any difference of HRQoL between groups. Nonparametric measures and the Quade ANCOVA^31^ were performed when data normality or assumptions of ANCOVA was violated.

The effect of loss of following on the study findings was accounted for by multiple imputation.^32^ Missing values of study outcomes at postweaning follow-ups were imputed under the assumption that missing outcome measures can be explained by covariates at baseline including major curve Cobb angle, curve type, and skeletal maturity. Pooled data from 17 imputations (based on loss of follow-up rate at postweaning 24 months and 5% variation coefficient) were analyzed in primary and secondary analyses.

All analyses were performed using SPSS version 28.0 (IBM). Statistical significance was set at a Bonferroni-adjusted 2-tailed *P* < .008 for intergroup comparisons of primary study outcome and secondary study outcome of truncal balance, while HRQoL measures and other radiological parameters were subjected to adjusted *P* < .002 in accordance with their multiplicity.

## Results

### Baseline Characteristics

**Figure 1.**
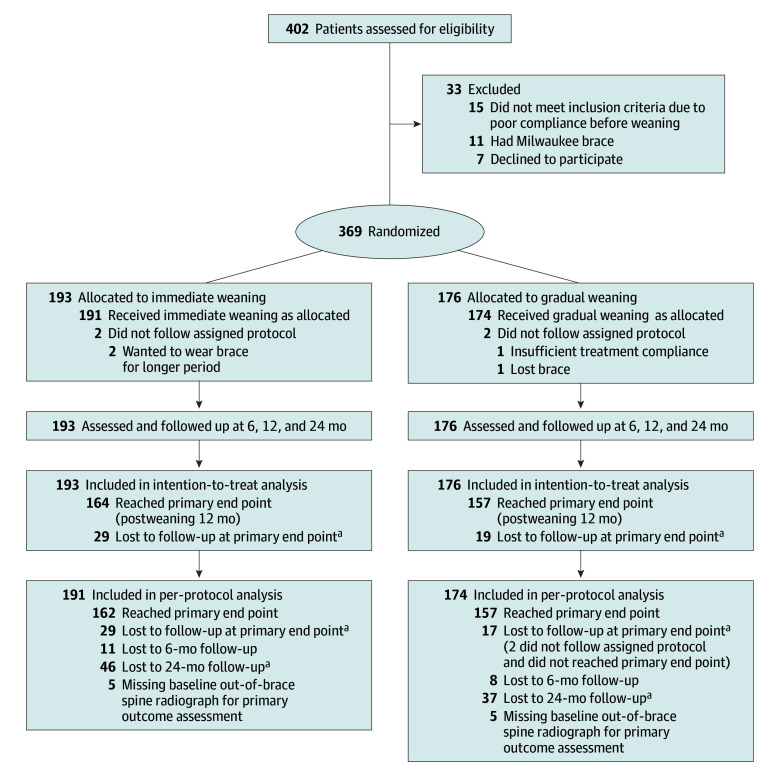
Flowchart of Patient Recruitment ^a^The follow-up time point occurred during the COVID-19 pandemic, during which time clinical service was restricted.

A total of 369 patients (mean [SD] age, 14.9 [1.1] years; 304 [83.4%] girls) with AIS were recruited and randomized to immediate weaning (n = 193) and gradual weaning (n = 176). The PP subset consisted of 365 patients (191 in the gradual weaning group and 174 in the immediate weaning group) (Figure 1). There were 284 patients (137 in gradual weaning and 147 in immediate weaning) who returned at 24-month follow-up. The gradual weaning group had a mean (SD) 9.0 (3.5) hours per day of brace wear. Table 1 presents patient baseline characteristics, which were comparable between groups.

**Table 1.** Baseline Characteristics and of Immediate and Gradual Weaning Groups at the Time of Weaning

Characteristic	ITT population (n = 369), No. (%)				PP population (n = 365)			
	No.	Immediate weaning, No. (%)	No.	Gradual weaning, No. (%)	No.	Immediate weaning, No. (%)	No.	Gradual weaning, No. (%)
Sex								
Female	193	156 (80.8)	176	148 (84.1)	193	155 (81.2)	174	146 (83.9)
Male		37 (19.2)		28 (15.9)		36 (18.8)		28 (16.1)
Curve type								
Thoracic	193	38 (19.7)	176	43 (24.4)	193	38 (19.9)	174	43 (24.7)
Thoracolumbar/lumbar		155 (80.3)		133 (75.6)		153 (80.1)		131 (75.3)
Cobb angle at weaning, mean (SD),degrees	193							
Major curve	145	30.1 (7.8)	176	29.3 (8.4)	191	30.2 (7.8)	174	29.3 (8.4)
Minor curve^a^	193	24.3 (7.6)	137	23.4 (8.0)	144	24.3 (7.6)	136	23.3 (8.1)
Duration of bracing, mean (SD), mo	193	26.7 (12.8)	176	25.0 (12.7)	191	26.8 (12.8)	174	25.1 (12.7)
**Girls**								
Age at weaning, mean (SD), y	156	14.8 (1.0)	148	14.5 (0.9)	155	14.8 (1.0)	146	14.6 (0.9)
Standing height, mean (SD), cm	156	160.1 (5.5)	148	160.4 (6.3)	155	160.1 (5.5)	146	160.3 (6.2)
Sitting height, mean (SD), cm	156	86.9 (2.8)	148	87.1 (3.1)	155	86.9 (2.8)	146	87.1 (3.2)
Arm span, mean (SD), cm	156	159.6 (6.7)	148	159.3 (7.2)	155	159.6 (6.7)	146	159.3 (7.2)
Weight, mean (SD), kg	156	47.7 (5.9)	148	47.3 (6.1)	155	47.7 (5.9)	146	47.3 (6.1)
BMI, mean (SD)	156	18.6 (2.0)	148	18.4 (2.1)	155	18.6 (2.0)	146	18.4 (2.1)
Time postmenarche (remaining patients were premenarche), mean (SD), mo	132	30.3 (7.6)	124	29.0 (6.4)	131	30.3 (7.7)	122	29.0 (6.3)
Skeletal maturity								
DRU classification								
R8	156	2 (1.3)	148	0	155	2 (1.3)	146	0
R9		33 (21.2)		36 (24.3)		33 (21.3)		36 (24.7)
R10		94 (60.3)		87 (58.8)		94 (60.6)		86 (58.9)
R11		27 (17.3)		25 (16.9)		26 (16.8)		24 (16.4)
U7		20 (12.8)		24 (16.2)		20 (12.9)		24 (16.4)
U8		93 (59.6)		81 (54.7)		92 (59.4)		80 (54.8)
U9		43 (27.6)		43 (29.1)		43 (27.7)		42 (28.8)
Sanders staging								
6	156	1 (0.6)	148	2 (1.4)	155	1 (0.6)	146	2 (1.4)
7A		20 (12.8)		24 (16.2)		20 (12.9)		24 (16.4)
7B		115 (73.7)		104 (70.3)		114 (73.5)		102 (69.9)
8		20 (12.8)		18 (12.2)		20 (12.9)		18 (12.3)
**Boys**								
Age at weaning, mean (SD), y	37	15.8 (1.4)	28	15.8 (1.0)	36	15.8 (1.4)	28	15.8 (1.0)
Standing height, mean (SD), cm	37	171.1 (6.5)	28	173.0 (7.0)	36	171.4 (6.3)	28	173.0 (7.0)
Sitting height, mean (SD), cm	37	91.6 (3.7)	28	92.5 (3.6)	36	91.9 (3.3)	28	92.5 (3.6)
Arm span, mean (SD), cm	37	171.7 (6.2)	28	174.2 (9.1)	36	172.0 (6.1)	28	174.2 (9.1)
Weight, mean (SD), kg	37	53.5 (7.9)	28	56.6 (12.1)	36	53.9 (7.7)	28	56.6 (12.1)
BMI, mean (SD)	37	18.3 (2.4)	28	18.8 (3.3)	36	18.3 (2.4)	28	18.8 (3.3)
Skeletal maturity								
DRU classification								
R9	37	6 (16.2)	28	4 (14.3)	36	5 (13.9)	28	4 (14.3)
R10		23 (62.2)		20 (71.4)		23 (63.9)		20 (71.4)
R11		8 (21.6)		4 (14.3)		8 (22.2)		4 (14.3)
U7		3 (8.1)		5 (17.9)		2 (5.6)		5 (17.9)
U8		22 (59.5)		15 (53.6)		22 (61.1)		15 (53.6)
U9		12 (32.4)		8 (28.6)		12 (33.3)		8 (28.6)
Sanders staging								
6	37	1 (2.7)	28	1 (3.6)	36	0	28	1 (3.6)
7A		2 (5.4)		6 (21.4)		2 (5.6)		6 (21.4)
7B		27 (73.0)		18 (64.3)		27 (75.0)		18 (64.3)
8		7 (18.9)		3 (10.7)		7 (19.4)		3 (10.7)

Abbreviations: BMI, body mass index (calculated as weight in kilograms divided by height in meters squared); DRU, distal radius and ulna; ITT, intention to treat; PP, per protocol; R, radius grade; U, ulna grade.

^a^ Number of patients analyzed for minor curves only when minor curves were present.

### Primary Outcome

**Figure 2.**
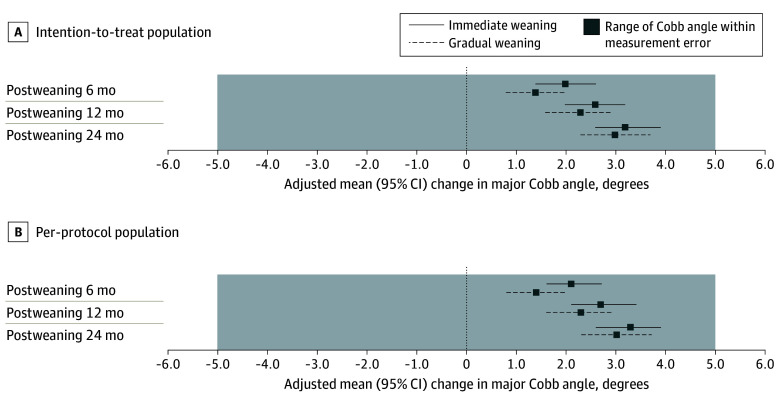
Primary Study Outcome at 6, 12, and 24 Months Postweaning

In both the ITT and PP populations, primary analyses found no significant difference in changes of major and minor Cobb angles between the 2 protocols, except for only minor Cobb angle change at postweaning 6 months. Secondary analyses similarly found no significant intergroup differences in major and minor Cobb angle changes with covariate adjustment (Table 2). At postweaning 24 months, the adjusted mean change of major Cobb angle was 3.2° (95% CI, 2.6 to 3.9) for immediate weaning and 3.0° (95% CI, 2.3 to 3.7) for gradual weaning in the ITT population, while adjusting for correlated covariates: major Cobb angle at weaning, DRU grades and Sanders stages at weaning, and curve type (Table 2). There were no significant differences in adjusted mean change of major Cobb angle between immediate and gradual weaning groups at postweaning 6 months (ITT: mean difference [MD], −0.6°; 95% CI, −1.4 to 0.2; *P* = .15; PP: MD, −0.7°; 95% CI, −1.6 to 0.1; *P* = .08), 12 months (ITT: MD, −0.4°; 95% CI, −1.3 to 0.5; *P* = .44; PP: MD, −0.5°; 95% CI, −1.4 to 0.4; *P* = .30), or 24 months (ITT: MD, −0.2°; 95% CI, −1.2 to 0.7; *P* = .64; PP: MD, −0.3°; 95% CI, −1.3 to 0.7; *P* = .56) (Figure 2). The only significant difference was in the gradual weaning group having smaller minor Cobb angle change with covariates at postweaning 6 months (ITT: MD, −1.5°; 95% CI, −2.5 to −0.4; *P* = .007) or without. The effect of weaning protocol on major Cobb angle changes at postweaning 6, 12, and 24 months was insignificant, with or without covariate adjustment. This rejects the null hypothesis that gradual weaning was better in Cobb angle maintenance than immediate weaning. Analysis of the unimputed primary study outcome demonstrated similar results (eTable 4 in Supplement 2).

**Table 2.** Intergroup Comparison of Primary Outcome in Primary Analysis Without Adjustment and Secondary Analyses With Covariate Adjustment

Postweaning time point	No.				Mean difference (95% CI)^a^	Statistic	Partial η^2^	*P* value
	Immediate weaning		Gradual weaning					
**Primary analyses**								
**ITT population**								
Change of Cobb angle of major curve (vs baseline), mean (SD), degrees								
6 mo	193	2.0 (3.9)	176	1.4 (4.1)	−0.6 (−1.4 to 0.2)	*t*, −1.286	NA	.17
12 mo	193	2.6 (4.6)	176	2.3 (4.2)	−0.3 (−1.2 to 0.6)	*t*, −0.565	NA	.47
24 mo	193	3.2 (4.7)	176	3.0 (4.9)	−0.3 (−1.2 to 0.7)	*t*, −0.529	NA	.60
Change of Cobb angle of minor curve (vs baseline), mean (SD), degrees^b^								
6 mo	136	1.8 (4.4)	135	0.3 (4.3)	−1.5 (−2.5 to −0.4)	*t*, −2.775	NA	.006^c^
12 mo	136	2.3 (4.6)	135	1.3 (4.6)	−1.0 (−2.1 to 0.1)	*t*, −1.717	NA	.11
24 mo	136	1.9 (4.5)	135	1.0 (4.5)	−0.9 (−2.0 to 0.2)	*t*, −1.593	NA	.17
**PP**								
Change of Cobb angle of major curve (vs baseline), mean (SD), degrees								
6 mo	186	2.1 (3.8)	169	1.4 (4.0)	−0.7 (−1.5 to 0.1)	*t*, −1.657	NA	.10
12 mo	186	2.7 (4.4)	169	2.3 (4.2)	−0.4 (−1.3 to 0.5)	*t*, −0.959	NA	.34
24 mo	186	3.3 (4.5)	169	3.0 (4.8)	−0.3 (−1.3 to 0.7)	*t*, −0.637	NA	.52
Change of Cobb angle of minor curve (vs baseline), mean (SD), degrees^b^								
6 mo	132	1.8 (4.5)	130	0.4 (4.2)	−1.4 (−2.5 to -0.4)	*t*, −2.632	NA	.009
12 mo	132	2.3 (4.9)	130	1.4 (4.5)	−0.9 (−2.1 to 0.4)	*t*, −1.405	NA	.16
24 mo	132	1.9 (5.2)	130	1.1 (5.6)	−0.8 (−2.0 to 0.5)	*t*, −1.190	NA	.24
**Secondary analyses**								
**ITT**								
Change of Cobb angle of major curve (vs baseline), adjusted mean (95% CI) [SE], degrees								
6 mo	193	2.0 (1.4 to 2.6) [0.3]	176	1.4 (0.8 to 2.0) [0.3]	−0.6 (−1.4 to 0.2)	*F*, 2.078	0.006	.15
12 mo	193	2.6 (2.0 to 3.2) [0.3]	176	2.3 (1.6 to 2.9) [0.3]	−0.4 (−1.3 to 0.5)	*F*, 0.607	0.002	.44
24 mo	193	3.2 (2.6 to 3.9) [0.3]	176	3.0 (2.3 to 3.7) [0.4]	−0.2 (−1.2 to 0.7)	*F*, 0.225	0.001	.64
Change of Cobb angle of minor curve (vs baseline), adjusted mean (95% CI) [SE], degrees^b^								
6 mo	136	1.8 (1.0 to 2.5) [0.4]	135	0.3 (-0.4 to 1.1) [0.4]	−1.5 (−2.5 to -0.4)	*F*, 7.461	0.028	.007^c^
12 mo	136	2.3 (1.5 to 3.1) [0.4]	135	1.4 (0.6 to 2.1) [0.4]	−1.0 (−2.1 to 0.2)	*F*, 2.820	0.011	.09
24 mo	136	1.9 (1.1 to 2.7) [0.4]	135	1.1 (0.3 to 1.8) [0.4]	−0.8 (−1.9 to 0.3)	*F*, 2.227	0.008	.14
**PP**								
Change of Cobb angle of major curve (vs baseline), adjusted mean (95% CI) [SE], degrees								
6 mo	186	2.1 (1.6 to 2.7) [0.3]	169	1.4 (0.8 to 2.0) [0.3]	−0.7 (−1.6 to 0.1)	*F*, 3.115	0.009	.08
12 mo	186	2.7 (2.1 to 3.4) [0.3]	169	2.3 (1.6 to 2.9) [0.3]	−0.5 (−1.4 to 0.4)	*F*, 1.088	0.003	.30
24 mo	186	3.3 (2.6 to 3.9) [0.3]	169	3.0 (2.3 to 3.7) [0.4]	−0.3 (−1.3 to 0.7)	*F*, 0.336	0.001	.56
Change of Cobb angle of minor curve (vs baseline), adjusted mean (95% CI) [SE], degrees^b^								
6 mo	132	1.8 (1.1 to 2.5) [0.4]	130	0.4 (-0.4 to 1.1) [0.4]	−1.4 (−2.5 to −0.4)	*F*, 6.936	0.026	.01
12 mo	132	2.3 (1.4 to 3.1) [0.4]	130	1.4 (0.5 to 2.3) [0.4]	−0.9 (−2.0 to 0.3)	*F*, 2.237	0.009	.17
24 mo	132	1.8 (0.9 to 2.7) [0.5]	130	1.1 (0.2 to 2.1) [0.5]	−0.7 (−1.9 to 0.4)	*F*, 1.640	0.006	.27

Abbreviations: *F*, *F* ratio adjusted for covariates (major curve Cobb angles, curve types, and bone age at weaning) in analysis of covariance for secondary analyses; ITT, intention to treat; NA, not applicable for primary analyses; PP, per protocol; *t*, *t* statistic value for independent-samples *t* test with the Levene test for primary analyses.

^a^ Gradual weaning minus immediate weaning. Mean difference for primary analyses; mean difference based on adjusted estimated marginal means for secondary analyses.

^b^ Number of patients analyzed for minor curves only when minor curves were present, hence not subjected to multiple imputation.

^c^ Statistical significance at Bonferroni-adjusted *P *< .008.

### Subgroup Analyses

At postweaning 24 months, 284 patients (77.0%) in the ITT population returned for follow-up. Patients with or without 24-month follow-up demonstrated no significant differences in baseline characteristics (eTable 5 in Supplement 2). There were 54 of 284 patients (19.0%) in the ITT population and 53 of 282 (18.8%) in the PP population (29 in the gradual weaning group and 24 in the immediate weaning group) who experienced curve progression, while 30 of 282 in the PP population (10.6%) demonstrated rebound (11 in gradual weaning and 19 in immediate weaning). The counts of progression, nonprogression, and rebound cases between weaning protocols were not significantly different (χ^2^_2_ = 2.123; *P* = .35). Major Cobb angles at weaning were larger for those who progressed vs those who did not in each protocol (mean [SD], immediate: 35.7° [7.2] vs 29.8° [8.3]; MD, 5.9°; 95% CI, 2.2 to 9.6°; *P* = .002; gradual: 33.5° [6.6] vs 28.5° [8.5]; MD, 5.0°; 95% CI, 1.4 to 8.6; *P* = .006). Curve progression with skeletal maturity status at weaning were examined (eTable 6 in Supplement 2). Weaning protocol was not associated with curve progression in multivariable regressions with: radius grades χ^2^_5_ = 34.444; *P* < .001; ulnar grades χ^2^_5_ = 39.329; *P* < .001; Sanders stages χ^2^_6_ = 36.606; *P* < .001; Risser stages χ^2^_5_ = 24.128; *P* < .001. Major Cobb angle (β, 0.10; 95% CI, 0.06-0.14; *P* < .001) and skeletal maturity at weaning (radius grades: Wald χ^2^_2_ = 9.854; *P* = .007; ulna grades: Wald χ^2^_2_ = 13.292; *P* = .001; Sanders stages: Wald χ^2^_3_ = 12.419; *P* = .006; except Risser stages: Wald χ^2^_2_ = 1.498; *P* = .47) were associated with curve progression (eTable 7 in Supplement 2).

### Secondary Outcomes

In both the ITT and PP populations, changes in truncal balance were not significantly different between groups at postweaning 6, 12, and 24 months with (Table 3) and without (eTable 8a in Supplement 2) covariate adjustment. Intergroup differences of other radiological parameters were also insignificant (eTable 8b in Supplement 2).

**Table 3.** Intergroup Comparison of Secondary Outcomes (Change of Truncal Balance and Health-Related Quality of Life Measures) With Covariate Adjustment

Postweaning time point	No.				Mean difference (95% CI)^a^	*F*	Partial η^2^	*P* value^b^
	Immediate weaning		Gradual weaning					
**Truncal balance**								
**ITT**								
Change of truncal shift (vs baseline) to adjusted mean (SE) to mm								
6 mo	193	0.9 (0.6)	176	1.1 (0.6)	0.2 (−1.5 to 1.9)	0.057	0.000	.81
12 mo	193	1.4 (0.6)	176	2.3 (0.6)	0.9 (−0.9 to 2.6)	0.936	0.003	.33
24 mo	193	2.5 (0.7)	176	2.3 (0.7)	−0.2 (−2.1 to 1.7)	0.045	0.000	.83
Change of listing (vs baseline) to adjusted mean (SE) to mm								
6 mo	193	0.5 (0.7)	176	0.7 (0.7)	0.2 (−1.8 to 2.1)	0.033	0.000	.86
12 mo	193	0.2 (0.7)	176	1.1 (0.7)	0.9 (−1.1 to 2.9)	0.755	0.002	.39
24 mo	193	2.1 (0.8)	176	2.2 (0.8)	0.1 (−2.1 to 2.3)	0.006	0.000	.94
**PP**								
Change of truncal shift (vs baseline) to adjusted mean (SE) to mm								
6 mo	186	0.8 (0.6)	169	1.3 (0.6)	0.5 (−1.2 to 2.1)	0.344	0.001	.56
12 mo	186	1.3 (0.6)	169	2.5 (0.6)	1.2 (−0.5 to 3.0)	1.941	0.006	.16
24 mo	186	2.3 (0.7)	169	2.5 (0.7)	0.2 (−1.7 to 2.1)	0.036	0.000	.85
Change of listing (vs baseline) to adjusted mean (SE) to mm								
6 mo	186	0.5 (0.7)	169	0.7 (0.7)	0.3 (−1.7 to 2.2)	0.072	0.000	.79
12 mo	186	-0.1 (0.7)	169	1.2 (0.8)	1.4 (−0.7 to 3.4)	1.721	0.005	.19
24 mo	186	1.9 (0.8)	169	2.4 (0.8)	0.4 (−1.8 to 2.7)	0.138	0.000	.71
**HRQoL measures**								
**ITT**								
SRS-22r domain scores, median (IQR)								
Function								
6 mo	193	5.00 (4.80-5.00)	176	5.00 (4.80-5.00)	NA	1.562	0.005	.21
12 mo	193	5.00 (4.80-5.00)	176	5.00 (4.85-5.00)	NA	0.103	0.001	.75
24 mo	193	5.00 (4.00-5.00)	176	5.00 (4.80-5.00)	NA	1.682	0.005	.20
Pain								
6 mo	193	4.90 (4.60-5.00)	176	4.80 (4.60-5.00)	NA	0.613	0.001	.43
12 mo	193	5.00 (4.60-5.00)	176	5.00 (4.60-5.00)	NA	0.005	0.000	.95
24 mo	193	4.80 (4.50-5.00)	176	4.80 (4.50-5.00)	NA	0.392	0.001	.53
Self-image								
6 mo	193	4.00 (3.60-4.50)	176	3.80 (3.40-4.40)	NA	4.129	0.006	.04
12 mo	193	4.20 (3.60-4.60)	176	4.10 (3.70-4.50)	NA	0.199	0.000	.66
24 mo	193	4.00 (3.60-4.35)	176	4.10 (3.75-4.45)	NA	2.542	0.005	.11
Mental health								
6 mo	193	4.40 (4.00-5.00)	176	4.40 (4.00-5.00)	NA	0.008	0.000	.93
12 mo	193	4.40 (4.00-4.80)	176	4.60 (4.00-5.00)	NA	0.892	0.003	.35
24 mo	193	4.20 (4.00-4.70)	176	4.40 (4.00-4.80)	NA	1.078	0.002	.30
Satisfaction with treatment^c^								
6 mo	76	4.00 (3.50-4.50)	110	4.00 (4.00-4.50)	NA	0.407	0.004	.52
12 mo	63	4.00 (3.50-4.50)	81	4.00 (4.00-4.50)	NA	0.071	0.000	.79
24 mo	74	4.00 (3.50-4.50)	71	4.00 (4.00-4.50)	NA	0.060	0.000	.81
SRS-22r total score, median (IQR)								
6 mo	193	4.50 (4.30-4.74)	176	4.45 (4.24-4.68)	NA	2.732	0.005	.10
12 mo	193	4.55 (4.34-4.75)	176	4.50 (4.30-4.75)	NA	0.030	0.000	.86
24 mo	193	4.45 (4.31-4.60)	176	4.50 (4.35-4.65)	NA	0.707	0.003	.40
EQ-5D utility score, median (IQR)								
6 mo	193	1.000 (0.933-1.000)	176	1.000 (0.950-1.000)	NA	0.024	0.001	.88
12 mo	193	1.000 (0.930-1.000)	176	1.000 (0.948-1.000)	NA	0.040	0.001	.84
24 mo	193	1.000 (1.000-1.000)	176	1.000 (1.000-1.000)	NA	0.003	0.000	.96
EQ-VAS score, median (IQR)								
6 mo	193	88.0 (80.0-95.0)	176	90.0 (80.0-95.0)	NA	0.118	0.001	.73
12 mo	193	90.0 (80.0-95.0)	176	90.0 (80.0-95.0)	NA	0.158	0.002	.69
24 mo	193	87.0 (80.0-93.0)	176	86.0 (80.0-95.0)	NA	0.085	0.001	.77
**PP**								
SRS-22r domain score, median (IQR), function								
6 mo	186	5.00 (4.80-5.00)	169	5.00 (4.80-5.00)	NA	1.970	0.006	.16
12 mo	186	5.00 (4.85-5.00)	169	5.00 (4.90-5.00)	NA	0.009	0.001	.92
24 mo	186	5.00 (4.90-5.00)	169	5.00 (4.80-5.00)	NA	1.780	0.006	.18
Pain								
6 mo	186	5.00 (4.60-5.00)	169	4.80 (4.60-5.00)	NA	0.515	0.002	.47
12 mo	186	5.00 (4.60-5.00)	169	5.00 (4.60-5.00)	NA	0.001	0.000	.98
24 mo	186	4.80 (4.50-5.00)	169	4.80 (4.60-5.00)	NA	0.155	0.001	.69
Self-image								
6 mo	186	4.10 (3.60-4.45)	169	3.80 (3.40-4.40)	NA	4.788	0.011	.03
12 mo	186	4.20 (3.70-4.55)	169	4.00 (3.60-4.45)	NA	0.453	0.002	.50
24 mo	186	4.00 (3.60-4.35)	169	4.10 (3.75-4.50)	NA	1.663	0.004	.20
Mental health								
6 mo	186	4.40 (4.00-5.00)	169	4.40 (4.00-5.00)	NA	0.089	0.000	.77
12 mo	186	4.40 (4.00-4.80)	169	4.60 (4.00-5.00)	NA	0.733	0.001	.39
24 mo	186	4.30 (4.00-4.70)	169	4.40 (4.00-4.80)	NA	0.866	0.004	.35
Satisfaction with treatment^c^								
6 mo	72	4.00 (3.50-4.50)	108	4.00 (4.00-4.50)	NA	0.218	0.001	.64
12 mo	59	4.00 (4.00-4.50)	79	4.00 (4.00-4.50)	NA	0.250	0.001	.62
24 mo	71	4.00 (3.50-4.50)	70	4.00 (4.00-4.50)	NA	0.066	0.001	.80
SRS-22r total score, median (IQR)								
6 mo	186	4.50 (4.32-4.74)	169	4.45 (4.24-4.68)	NA	3.262	0.006	.07
12 mo	186	4.55 (4.35-4.75)	169	4.50 (4.30-4.75)	NA	0.030	0.000	.86
24 mo	186	4.50 (4.33-4.60)	169	4.50 (4.36-4.65)	NA	0.715	0.002	.40
EQ-5D utility score, median (IQR)								
6 mo	186	1.000 (0.983-1.000)	169	1.000 (1.000-1.000)	NA	0.016	0.001	.90
12 mo	186	1.000 (0.930-1.000)	169	1.000 (1.000-1.000)	NA	0.002	0.001	.96
24 mo	186	1.000 (1.000-1.000)	169	1.000 (1.000-1.000)	NA	0.001	0.001	.98
EQ-VAS score, median (IQR)								
6 mo	186	88.0 (80.0-95.0)	169	90.0 (80.0-95.0)	NA	0.002	0.000	.96
12 mo	186	90.0 (80.0-95.0)	169	90.0 (80.0-96.0)	NA	0.222	0.001	.64
24 mo	186	87.0 (80.0-94.0)	169	88.0 (80.0-95.0)	NA	0.129	0.000	.72

Abbreviations: EQ-5D, EuroQol 5-dimension; EQ-VAS, EuroQol visual analogue scale; *F*, *F* ratio adjusted for covariates (major curve Cobb angles, curve types, and bone age at weaning) for analysis of covariance for truncal balance or *F* ratio adjusted for covariates (major curve Cobb angles, curve types, and bone age at weaning) for the Quade analysis of covariance for HRQoL measures; HRQoL, health-related quality of life; ITT, intention to treat; NA, not applicable for the Quade analysis of covariance (based on ranks); PP, per protocol; SRS-22r, refined Scoliosis Research Society 22-item.

^a^ Gradual weaning minus immediate weaning; based on adjusted estimated marginal means.

^b^ Statistical significance at Bonferroni-adjusted *P* < .008 for truncal balance parameters and statistical significance at Bonferroni-adjusted *P* < .002 for HRQoL measures.

^c^ Patients could opt for answering questions for this domain if without treatment but not affecting calculation of the SRS total score, hence not subjected to multiple imputation.

The 2 protocols had no significant differences of SRS-22r total scores, EQ-5D utility scores, or EQ-VAS scores at postweaning 6, 12, and 24 months (Table 3). At postweaning 24 months, gradual and immediate weaning groups had similar SRS-22r total scores (median [IQR], 4.50 [4.35-4.65] vs 4.45 [4.31-4.60]; *P* = .40), EQ-5D utility scores (median [IQR], 1.000 [1.000-1.000] vs 1.000 [1.000-1.000]; *P* = .96), and EQ-VAS scores (median [IQR], 86.0 [80.0-95.0] vs 87.0 [80.0-93.0]; *P* = .77) with covariate adjustment in the ITT population and similarly in PP population. SRS-22r pain domain scores were comparable at all follow-up points in the ITT population (24-month median [IQR] gradual vs immediate weaning, 4.80 [4.50-5.00] vs 4.80 [4.50-5.00]; *P* = .53) and the PP population (Table 3), with or without covariate adjustment (eTable 9 in Supplement 2).

## Discussion

This randomized clinical trial aimed to address the lack of consensus on whether it is best to adopt the method of gradual vs immediate brace removal in AIS. It is important to avoid prolonged bracing as brace wear is potentially associated with flatback,^33^ muscle weakness,^13^ and reduced HRQoL with time.^34,35^ On the other hand, the outcomes of bracing, especially curve magnitude and balance, need to be maintained without deterioration after brace removal.

In this study, the changes in major Cobb angle were found similar at postweaning 6, 12, and 24 months between immediate weaning and gradual weaning protocols in AIS. The magnitude of Cobb angle changes from baseline for each weaning protocol was within the measurement error range of 5° (Figure 2), while the intergroup difference in change of Cobb angle was within the equivalence margin of 2°. Such intergroup comparisons were valid given both major and minor Cobb angles at weaning were comparable. For the secondary outcome, both weaning protocols demonstrated similar changes in truncal balance after weaning given an average of 9.0 hours per day of nocturnal brace compliance by the gradual weaning group. Similar results were found with or without adjustment for correlated covariates. Through the 2 complementary strategies^29^ in data analyses—that is, according to the ITT principle and PP analysis—we ensured the advantage of randomization was maintained in the PP population.^36^ Concordant results of primary and secondary outcomes in both ITT and PP populations at all 3 postweaning time points showed no significant difference between immediate and gradual weaning protocols. The gradual weaning group did not reach the predefined criteria for superiority.

The counts of curve progression after weaning were similar for the 2 weaning protocols. Among curve progression cases, we differentiated between those who experienced true progression from rebound (loss of curve correction but progression within 5° from prebrace major Cobb angle), with no significant intergroup difference in the counts for progression, regression, or rebound (eFigure in Supplement 2). Patients with postweaning curve progression were those with significantly larger major Cobb angle at weaning regardless of gradual or immediate weaning protocol. Weaning protocol was not associated with postweaning curve progression, whereas major Cobb angle and DRU and Sanders stages at weaning were significant factors. Hence, curve magnitude and remaining growth potential at weaning had the greatest influence on postweaning curve progression risk.^37,38,39,40,41^ Larger weaning Cobb angle and less mature gradings carry higher odds of curve progression. Despite the fact that all patients had reached Risser stage 4 or greater at weaning, the corresponding DRU and Sanders stages ranged from R8 to R11, U7 to U9, and SS6 to SS8, with less mature grades like R9 or less or U7 having greater odds ratio of curve progression. These echo the findings of postweaning curve progression in Cheung et al.^28^ Risser staging at weaning lacked association with whether curve progressed or not, this coincides with previous findings that Risser staging alone was unable to differentiate patients with risk of curve progression.^28,42^

We hypothesized that gradual weaning may be superior with better Cobb angle and truncal balance maintenance, and possible gradual adjustment of paraspinal musculature to the braceless environment. Gradual weaning may help minimize muscle discomfort from sudden straining without the brace, whereas immediate weaning is thought to lead to more back pain. Yet, both weaning protocols demonstrated comparable HRQoL, including not only overall but specific aspects, like pain, as captured by the SRS-22r pain domain and EQ-5D pain/discomfort dimension. SRS-22r pain domain scores were not significantly different between immediate and gradual weaning at all follow-up points. Any postweaning intergroup difference was likely within the clinically relevant, minimum detectable measurement difference of 0.3^42^ which allows comparison between study groups,^43^ and also within the minimum clinically important difference of 0.98.^44^ However, both minimum detectable measurement difference and minimum clinically important difference of pain domain scores were only defined in surgically treated but not braced AIS cohorts. Apart from the SRS-22r total score, overall quality of life reflected by EQ-5D utility score and EQ-VAS score were also similar between the 2 protocols; any EQ-5D utility score difference did not reach the value of .05 as detected previously between patient groups.^10^

### Limitations

This trial lacks immediate postweaning 1- to 2-week assessment of any muscle pain or paraspinal muscle adjustment, as short-interval returns were not possible. We therefore cannot conclude on any difference of muscle pain level between weaning protocols though no difference of HRQoL was reflected at 6-month follow-up. Also, postweaning exercise and activity levels could have differed between groups and that could have had an effect on the strength of spinal muscles relating to truncal balance and HRQoL. In addition, the discrepancy of group sample size is suboptimal, and it could have been avoided.

## Conclusions

As gradual brace weaning did not demonstrate superiority with predefined criteria of maintenance of Cobb angle and truncal balance after brace weaning, this clinical trial reveals that gradual weaning does not have the perceived advantage over immediate weaning. Immediate weaning can achieve similar maintenance of brace outcomes as well as HRQoL in AIS. We recommend the more frequent use of immediate weaning protocol with appropriate patient selection, as patients with immediate weaning can return to increased exercises and activity level at an earlier time. The prognostication of curve progression after brace weaning is dependent on curve magnitude and skeletal maturity status at weaning, instead of the way brace wear is weaned. Current practice based on Risser staging can possibly be improved by new brace weaning protocol using other skeletal maturity indices such as Sanders staging and DRU classification.

## Notes

Supplement 1. Trial protocol

Supplement 2. eTable 1. Baseline profile of immediate and gradual weaning group eTable 2. Correlation of baseline parameters with changes of major curve Cobb angles for ITT population eTable 3. Correlation of baseline parameters with changes of truncal balance and other radiological parameters and health-related quality of life measures for intention-to- treat (ITT) population eTable 4. Intergroup comparison of primary outcome without imputation in (a) primary analysis without adjustment and (b) secondary analyses with covariate adjustment eTable 5. Comparison of baseline demographics and patient characteristics at the time of brace weaning for all randomized patients with and without ITT post-weaning 24- month follow-up eTable 6. Cross-tabulation of skeletal maturity status at weaning and brace outcomes of curve progression at post-weaning 24-months between immediate and gradual weaning protocols in the Intention-to-treat (ITT) and per-protocol (PP) population eTable 7. Logistic regression for curve progression at post-weaning 24 months for the per-protocol (PP) population eTable 8. Intergroup comparison of (a) change of truncal balance without covariate adjustment and (b) other radiological parameters with/without covariate adjustment eTable 9. Comparison of health-related quality of life measures between groups without adjustment eFigure. Counts of non-progression, progression and rebound of curves at post- weaning 24 months for the PP population

Supplement 3. Data sharing statement

## References

[1] Katz DE, Durrani AA. Factors that influence outcome in bracing large curves in patients with adolescent idiopathic scoliosis. Spine (Phila Pa 1976). 2001;26(21):2354-2361. doi:10.1097/00007632-200111010-00012 11679821

[2] Maruyama T, Grivas TB, Kaspiris A. Effectiveness and outcomes of brace treatment: a systematic review. Physiother Theory Pract. 2011;27(1):26-42. doi:10.3109/09593985.2010.503989 21198404

[3] Rahman T, Bowen JR, Takemitsu M, Scott C. The association between brace compliance and outcome for patients with idiopathic scoliosis. J Pediatr Orthop. 2005;25(4):420-422. doi:10.1097/01.bpo.0000161097.61586.bb 15958887

[4] Weinstein SL, Dolan LA, Wright JG, Dobbs MB. Effects of bracing in adolescents with idiopathic scoliosis. N Engl J Med. 2013;369(16):1512-1521. doi:10.1056/NEJMoa1307337 24047455 PMC3913566

[5] Weinstein SL, Dolan LA, Spratt KF, Peterson KK, Spoonamore MJ, Ponseti IV. Health and function of patients with untreated idiopathic scoliosis: a 50-year natural history study. JAMA. 2003;289(5):559-567. doi:10.1001/jama.289.5.559 12578488

[6] Reamy BV, Slakey JB. Adolescent idiopathic scoliosis: review and current concepts. Am Fam Physician. 2001;64(1):111-116. 11456428

[7] Lonstein JE, Carlson JM. The prediction of curve progression in untreated idiopathic scoliosis during growth. J Bone Joint Surg Am. 1984;66(7):1061-1071. doi:10.2106/00004623-198466070-00013 6480635

[8] Landauer F, Wimmer C, Behensky H. Estimating the final outcome of brace treatment for idiopathic thoracic scoliosis at 6-month follow-up. Pediatr Rehabil. 2003;6(3-4):201-207. doi:10.1080/13638490310001636817 14713586

[9] Rogala EJ, Drummond DS, Gurr J. Scoliosis: incidence and natural history. a prospective epidemiological study. J Bone Joint Surg Am. 1978;60(2):173-176. doi:10.2106/00004623-197860020-00005 641080

[10] Cheung PWH, Wong CKH, Cheung JPY. An insight into the health-related quality of life of adolescent idiopathic scoliosis patients who are braced, observed, and previously braced. Spine (Phila Pa 1976). 2019;44(10):E596-E605. doi:10.1097/BRS.0000000000002918 31046000

[11] Noonan KJ, Dolan LA, Jacobson WC, Weinstein SL. Long-term psychosocial characteristics of patients treated for idiopathic scoliosis. J Pediatr Orthop. 1997;17(6):712-717. doi:10.1097/01241398-199711000-00004 9591971

[12] Vasiliadis E, Grivas TB, Savvidou O, Triantafyllopoulos G. The influence of brace on quality of life of adolescents with idiopathic scoliosis. Stud Health Technol Inform. 2006;123:352-356. 17108451

[13] Jafari Sarveolia A, Karimi M, Sharifmoradi K, Nadi A, Saljoughian P. The effect of Boston Brace on muscle length of patients with idiopathic scoliosis. Physical Treatments. 2015;5:163-170. https://ptj.uswr.ac.ir/article-1-264-en.html

[14] Eisinger DB, Kumar R, Woodrow R. Effect of lumbar orthotics on trunk muscle strength. Am J Phys Med Rehabil. 1996;75(3):194-197. doi:10.1097/00002060-199605000-00008 8663926

[15] Odermatt D, Mathieu PA, Beauséjour M, Labelle H, Aubin CE. Electromyography of scoliotic patients treated with a brace. J Orthop Res. 2003;21(5):931-936. doi:10.1016/S0736-0266(03)00038-X 12919883

[16] Li XF, Li H, Liu ZD, Dai LY. Low bone mineral status in adolescent idiopathic scoliosis. Eur Spine J. 2008;17(11):1431-1440. doi:10.1007/s00586-008-0757-z 18751741 PMC2583185

[17] Lu L, Dai Z, Lv G, Kang Y, Jiang Y. A novel therapeutic strategy for adolescent idiopathic scoliosis based on osteoporotic concept. Med Hypotheses. 2013;80(6):773-775. doi:10.1016/j.mehy.2013.03.008 23562283

[18] Cheung JP, Cheung PW, Samartzis D, Cheung KM, Luk KD. The use of the distal radius and ulna classification for the prediction of growth: peak growth spurt and growth cessation. Bone Joint J. 2016;98-b(12):1689-1696. doi:10.1302/0301-620X.98B12.BJJ-2016-0158.R1 27909133

[19] Kaelin AJ. Adolescent idiopathic scoliosis: indications for bracing and conservative treatments. Ann Transl Med. 2020;8(2):28. doi:10.21037/atm.2019.09.69 32055619 PMC6995912

[20] Cheung JPY, Cheung PWH, Shigematsu H, ; APSS Scoliosis Focus Group. Controversies with nonoperative management for adolescent idiopathic scoliosis: study from the APSS Scoliosis Focus Group. J Orthop Surg (Hong Kong). 2020;28(2):2309499020930291. doi:10.1177/2309499020930291 32529908

[21] World Medical Association. World Medical Association Declaration of Helsinki: ethical principles for medical research involving human subjects. JAMA. 2013;310(20):2191-2194. doi:10.1001/jama.2013.281053 24141714

[22] Rowe DE. The Scoliosis Research Society brace manual. Accessed December 10, 2023. https://www.srs.org/Education/Manuals-and-Presentations/SRS-Bracing-Manual

[23] Negrini S, Donzelli S, Aulisa AG, . 2016 SOSORT guidelines: orthopaedic and rehabilitation treatment of idiopathic scoliosis during growth. Scoliosis Spinal Disord. 2018;13:3. doi:10.1186/s13013-017-0145-8 29435499 PMC5795289

[24] Risser JC. The Iliac apophysis; an invaluable sign in the management of scoliosis. Clin Orthop. 1958;11(11):111-119. 13561591

[25] Cheung JP, Samartzis D, Cheung PW, Cheung KM, Luk KD. Reliability analysis of the distal radius and ulna classification for assessing skeletal maturity for patients with adolescent idiopathic scoliosis. Global Spine J. 2016;6(2):164-168. doi:10.1055/s-0035-1557142 26933618 PMC4771512

[26] Sanders JO, Browne RH, McConnell SJ, Margraf SA, Cooney TE, Finegold DN. Maturity assessment and curve progression in girls with idiopathic scoliosis. J Bone Joint Surg Am. 2007;89(1):64-73. doi:10.2106/JBJS.F.00067 17200312

[27] Shi B, Guo J, Mao S, . Curve progression in adolescent idiopathic scoliosis with a minimum of 2 years’ follow-up after completed brace weaning with reference to the SRS standardized criteria. Spine Deform. 2016;4(3):200-205. doi:10.1016/j.jspd.2015.12.002 27927503

[28] Cheung JPY, Cheung PWH, Luk KD. When should we wean bracing for adolescent idiopathic scoliosis? Clin Orthop Relat Res. 2019;477(9):2145-2157. doi:10.1097/CORR.0000000000000781 31135558 PMC7000074

[29] Tripepi G, Chesnaye NC, Dekker FW, Zoccali C, Jager KJ. Intention to treat and per protocol analysis in clinical trials. Nephrology (Carlton). 2020;25(7):513-517. doi:10.1111/nep.13709 32147926

[30] Holmberg MJ, Andersen LW. Adjustment for baseline characteristics in randomized clinical trials. JAMA. 2022;328(21):2155-2156. doi:10.1001/jama.2022.21506 36394881

[31] Conover WJ, Iman RL. Analysis of covariance using the rank transformation. Biometrics. 1982;38(3):715-724. doi:10.2307/2530051 7171697

[32] Cheung JPY, Chong CHW, Cheung PWH. Underarm bracing for adolescent idiopathic scoliosis leads to flatback deformity: the role of sagittal spinopelvic parameters. Bone Joint J. 2019;101-b(11):1370-1378. doi:10.1302/0301-620X.101B11.BJJ-2019-0515.R1 31674249

[33] Cheung KM, Cheng EY, Chan SC, Yeung KW, Luk KD. Outcome assessment of bracing in adolescent idiopathic scoliosis by the use of the SRS-22 questionnaire. Int Orthop. 2007;31(4):507-511. doi:10.1007/s00264-006-0209-5 16896864 PMC2267629

[34] Piantoni L, Tello CA, Remondino RG, . Quality of life and patient satisfaction in bracing treatment of adolescent idiopathic scoliosis. Scoliosis Spinal Disord. 2018;13:26-26. doi:10.1186/s13013-018-0172-0 30564635 PMC6295031

[35] Chan AW, Tetzlaff JM, Gøtzsche PC, . SPIRIT 2013 explanation and elaboration: guidance for protocols of clinical trials. BMJ. 2013;346:e7586. doi:10.1136/bmj.e7586 23303884 PMC3541470

[36] Kotwicki T, Chowanska J, Kinel E, Czaprowski D, Tomaszewski M, Janusz P. Optimal management of idiopathic scoliosis in adolescence. Adolesc Health Med Ther. 2013;4:59-73. doi:10.2147/AHMT.S32088 24600296 PMC3912852

[37] Grossman DC, Curry SJ, Owens DK, ; US Preventive Services Task Force. Screening for adolescent idiopathic scoliosis: US Preventive Services Task Force recommendation statement. JAMA. 2018;319(2):165-172. doi:10.1001/jama.2017.19342 29318284

[38] Sanders JO, Qiu X, Lu X, . The uniform pattern of growth and skeletal maturation during the human adolescent growth spurt. Sci Rep. 2017;7(1):16705. doi:10.1038/s41598-017-16996-w 29196711 PMC5711808

[39] Cheung JPY, Luk KD. Managing the pediatric spine: growth assessment. Asian Spine J. 2017;11(5):804-816. doi:10.4184/asj.2017.11.5.804 29093792 PMC5662865

[40] Luhmann S, Zaaroor-Regev D, Upasani VV, Shufflebarger H. The natural history of curve behavior after brace removal in adolescent idiopathic scoliosis: a literature review. Spine Deform. 2023;11(3):567-578. doi:10.1007/s43390-022-00638-x 36715866 PMC10147768

[41] Heegaard M, Tøndevold N, Dahl B, Andersen TB, Gehrchen M, Ohrt-Nissen S. Does Risser stage accurately predict the risk of curve progression in patients with adolescent idiopathic scoliosis treated with night-time bracing? Eur Spine J. 2023;32(9):3077-3083. doi:10.1007/s00586-023-07808-z 37314578

[42] Kelly MP, Lenke LG, Sponseller PD, . The minimum detectable measurement difference for the Scoliosis Research Society-22r in adolescent idiopathic scoliosis: a comparison with the minimum clinically important difference. Spine J. 2019;19(8):1319-1323. doi:10.1016/j.spinee.2019.04.008 30986576

[43] Chung AS, Copay AG, Olmscheid N, Campbell D, Walker JB, Chutkan N. Minimum clinically important difference: current trends in the spine literature. Spine (Phila Pa 1976). 2017;42(14):1096-1105. doi:10.1097/BRS.0000000000001990 27870805

[44] Carreon LY, Sanders JO, Diab M, Sucato DJ, Sturm PF, Glassman SD; Spinal Deformity Study Group. The minimum clinically important difference in Scoliosis Research Society-22 Appearance, Activity, and Pain domains after surgical correction of adolescent idiopathic scoliosis. Spine (Phila Pa 1976). 2010;35(23):2079-2083. doi:10.1097/BRS.0b013e3181c61fd7 20395881

