# Supplementary material for: Immediate vs Gradual Brace Weaning Protocols in Adolescent Idiopathic Scoliosis: A Randomized Clinical Trial
Source: JAMA Pediatr. 2024 Jun 3;178(7):657–68. doi: 10.1001/jamapediatrics.2024.1484 (PMC11148786; doi:10.1001/jamapediatrics.2024.1484)
Supplement: Supplement 1. — Trial protocol [file jamapediatr-e241484-s001.pdf]

Supplementary 1 Study Protocol

Table of Contents

Original Protocol	Pg. 1 - 17
Final Protocol	Pg. 19 - 35

STUDY PROTOCOL

A randomized controlled trial comparing gradual and immediate brace weaning for clinical management of adolescent idiopathic scoliosis patients

Protocol Number and Version

Version 1.0 dated 13 Feb 2017

Funding

Health and Medical Research Fund

1. INTRODUCTION

Scoliosi is a 3-dimensional deformity characterized as a lateral curvature of the spine in the coronal plane as measured by Cobb angle >10 degrees on plain radiographs. Patients with adolescent idiopathic scoliosis (AIS) may develop curve progression if left untreated, which can result in back pain, cosmetic disfigurement and early death from cardiopulmonary compromise.[1] The most common form of scoliosis is idiopathic and is usually detected during adolescence (10-18 years of age).[2] Based on the investigators' large-scale and internationally renowned Hong Kong-based scoliosis school screening program [3, 4], the prevalence of spine curvatures were as follows: ≥10 degrees was 2.2% for boys and 4.8% for girls; ≥20 degrees was 0.7% for boys and 2.8% for girls; and for ≥40 degrees the prevalence was 0.1% for boys and 0.4% for girls.[3] It is important to note that approximately only 78% of students in Hong Kong have participated in the school screening system. Hence, the prevalence may perhaps be an underestimation. Currently, in Hong Kong, all children diagnosed with AIS and noted to have a ≥20 degrees curve are referred to one of two scoliosis clinics for management as those who are skeletally immature have potential to deteriorate during growth and may be treated by bracing.[5] To place into context the magnitude of service required, "450-500 new braces" are prescribed per year in our clinic alone.

Bracing is the most common treatment method for controlling AIS progression and its main purpose is to prevent curves from reaching the surgical threshold of >50 degrees. However, prevention of any Cobb angle progression is also beneficial. Normally, at the end of skeletal growth, there is a significant reduction in risk of further curve progression. However, there is an increased risk of continued progression into adulthood of at least 1 degree per year for curvatures of ≥40 degrees at skeletal maturity. Hence, not only is a smaller Cobb angle at skeletal maturity more cosmetically acceptable, <u>preventing the 40 degree threshold can reduce the risk of adulthood deterioration and surgery</u> as suggested by natural history studies.[1, 6] Good brace compliance with more than 20 hours of daily use is proven to be effective in reducing curve progression.[7] High-level and established evidence have been published which support its efficacy.[8-10] Specifically, Weinstein et al[9] have demonstrated in a multicenter study that in patients with high-risk curves bracing significantly decreased curve progression and the need for surgery. Based on that study,

longer brace wearing decreased curve progression and the need for surgery.

At the end of skeletal growth, treatment is completed and braces are removed or "weaned".[7, 11, 12] This decision should be made in a timely fashion as drawbacks and complications have been noted with prolonged brace use. Prolonged bracing in children may reduce spinal mobility, lead to poor body image and self-esteem, and worsen quality of life.[13-18] There is a risk of osteoporosis and the prevalence of low bone mineral density in AIS patients may involve up to 38% of individuals.[19] A major reason why osteoporosis may occur is the reduced physical activity and use of core musculature due to brace immobilization. Although bracing improves trunk posture, it limits active body movement.[20] In the long-term, reduced spinal mobility and muscle endurance may occur up to 20 years after bracing has completed.[21] We currently have accurate parameters that help determine when patients reach skeletal maturity and no longer require further bracing.[22] However, **the manner in which brace treatment should be weaned is "clinician-dependent" and without any supporting evidence.**

For brace weaning, AIS patients can only rely on the surgeon's decision rather than evidence-based guidelines. This may be a **slow weaning process**, whereby the duration of brace wear is slowly reduced or changed to night-use only, or may be a **rapid immediate removal** of the brace. The main rationale for a slow gradual weaning process over a period of at least 6 months is to allow the spine to adapt to an environment without loading by the brace while maintaining the corrective posture. We postulate that by this method, the curve magnitude measured by the Cobb angle and the overall truncal balance and posture may be maintained even after brace removal. The resulting curve magnitude is the commonly used outcome measure of treatment as it has bearing on the likelihood of curve deterioration and surgery. However, truncal balance may be a larger concern for patients due to its larger impact on overall cosmesis. Whether this theory of gradual weaning benefits holds true is unknown but is supported by our knowledge of the spine's viscoelastic properties. The viscoelasticity of spinal structures including the ligaments, joint capsules and intervertebral discs allows deformation changes while loading and unloading the spine.[23] A lag-time response of spinal soft tissues to these changes have been demonstrated. Despite our limited knowledge on the effects of gradual weaning on the spine, deterioration in Cobb angle and truncal balance parameters may lead to worse cosmesis and quality of life, and possible need for surgery. Hence, **lack of an optimal brace weaning protocol is a significant**

limitation in our current management of AIS patients.

In this modern era, there is increasing emphasis placed on evidence-based health-care. Clinicians and health-care administrators are tasked to provide the best care for our patients. While some clinicians may terminate brace treatment with a gradual weaning period, others are content with immediate brace removal to avoid prolonged brace treatment. Due to the lack of evidence for either method, it is an appropriate time to determine any advantages of gradual brace weaning for AIS patients. Bracing is a commonly used tool for preventing curve progression, and it is imperative to deliver the best treatment protocol to these children. With this purpose, we propose a randomized controlled trial to determine whether gradual weaning does benefit over immediate weaning.

2. STUDY OBJECTIVES

a. Primary Aim

To compare the degree of Cobb angle maintenance between immediate removal and gradual brace weaning protocols for AIS patients at 1 year after brace weaning.

Our hypothesis is that gradual brace weaning allows better adaptation to an unloaded environment without the brace in AIS patients, thereby resulting in better maintenance of the Cobb angle at 1 year after brace weaning.

b. Secondary Aim

i. To compare the degree of truncal balance maintenance between immediate removal and gradual brace weaning protocols for AIS patients after brace weaning.

We hypothesize that AIS patients with gradual brace weaning allows better adaptation to an unloaded environment without the brace thereby resulting with better maintenance of

truncal balance.

ii. To determine any differences in health-related quality of life (HRQOL) between immediate removal and gradual weaning protocols.

We hypothesize that due to improved Cobb angle and truncal balance maintenance, the HRQOL is better for the gradual weaning protocol.

3. STUDY DESIGN

a. Overall Study Design

This is an open-labelled, randomized controlled trial for testing two brace weaning protocols, namely immediate removal of brace to that of gradual brace weaning over a course of 6 months and followed-up for 12 months. The Clinical Trials Centre (CTC) at the University of Hong Kong is involved in reviewing the study design, statistical methodology of the concept, sample size calculation and will also aid in data and study site monitoring after study commencement.

b. Definition of Study Intervention

As there is no definition for "brace weaning", we adopt the protocol that our center has been using which has also been previously published.[24] Hence, we will be comparing two groups:

1) gradual brace weaning with the brace wearing time shortened to night wearing for 6 more months before stopping bracing altogether or

2) immediate brace weaning on the day of patient recruitment.

c. Study Duration

All subjects will be followed-up longitudinally up to 1 year after brace weaning has been issued. Therefore, this includes two clinic visits with one at 6 months to complete the gradual brace weaning protocol and another at 1 year to observe for any delayed changes to the overall curvature and balance, including loss of truncal balance and Cobb angle deterioration. This follow-up duration is based on previous work suggesting that the highest risk of post-brace weaning curve progression occurs within 6 months of stopping brace wear.[24] A 1-year collection of data is thus adequate for seeing differences between the two groups. This does not deviate significantly from our usual care as all AIS subjects in our clinic are followed-up at 6-monthly intervals and thus this duration of follow-up will be strictly followed and standardized. It is unlikely to experience drop-outs as these patients are under long-term follow-up in our department and will be recalled if they miss an appointment.

4. STUDY POPULATION

We will recruit AIS patients managed at the Duchess of Kent Children's Hospital (DKCH) in Hong Kong. DKCH is one of two only scoliosis centers in Hong Kong that manages AIS. Being under the same umbrella of the Hong Kong healthcare system, the data gathered at DKCH is representative of the whole of Hong Kong with regards to the same referral, treatment protocol and healthcare cost system.

a. Inclusion Criteria

A patient will be eligible for study participation if he/she meets all the following criteria:

- All AIS patients treated with underarm (Boston) bracing who are ready to undergo brace weaning
- Primary curve ranging between 20 and 40 degrees of Cobb angle measurement

- Have reached skeletal maturity based on the Scoliosis Research Society (SRS) standardized criteria: Risser stage ≥4, >2 years post-menarche, and no growth between 2 visits.

b. Exclusion Criteria

A patient will be excluded from the study if he/she meets any of the following criteria:

- Non-AIS patients
- Those undergoing Milwaukee bracing
- Patients who undergo brace weaning not because of skeletal maturity but because of the need for surgical intervention
- Cognitively impaired, unable to comply with study follow-up and protocol issued
- Refused consent for study.

5. RANDOMIAZATION AND ALLOCATION

Block randomization with randomly selected even number of block sizes will be used to ensure a balance in sample size between the study groups. A master randomization list will be generated by a statistician a priori. A web-based randomization application will be set up and the randomization list will be uploaded. Using the application, eligible patients who have consented to take part in the study will be randomly allocated to one of the two protocols by the project coordinator. The master randomization list will be managed independently by the project coordinator and concealed to all research staff until the study database closure. Attending specialist will perform patient recruitment and informed consent procedure. Project coordinator will be responsible for allocating recruited patients to either of the two arm of the trial.

6. STUDY ASSESSMENTS

All subjects will undergo weight, standing and sitting height, and arm span measurements, HRQOL and utility measurements, and whole spine standing posteroanterior (PA) and lateral radiographs at each clinic visit. The refined Scoliosis Research Society 22-item (SRS-22r) questionnaire and the EuroQoL 5-Dimension 5-Level (EQ5D) questionnaire will thus be provided at every visit for HRQOL and utility assessment. For all patients undergoing bracing, an orthotist assessment is also performed at each clinic visit for any brace modification and education. Further details regarding the measurements assessed are listed in the following pages.

Study Outcomes

a. Primary outcome

- Change in Cobb angle from baseline to 6 months and 1 year follow-up.

b. Secondary outcomes

- Change in truncal balance from baseline to 6 months and 1 year follow-up.
- SRS-22r total score at 6 months and 1 year follow-up.
- EQ-5D utility score at 6 months and 1 year follow-up.

Study Measurements Assessed

1. Socio-demographics

These data include the date of birth, gender, ethnic group, school grade, the number of siblings and siblings also under scoliosis management.

2. Body size and maturity parameters

This includes standing and sitting height, weight, and arm span at each follow-up

visit. All of these clinical parameters are measured routinely and hence, the height change since the previous follow-up can be measured. For girls, the menarche status and the start date of menarche will also be extracted. The Risser sign is determined by evaluating the extent of ossification of the iliac apophysis on the same PA spine radiographs used to assess the spine radiographic measurements. The bone age by the distal radius and ulna classification[22] will also be measured on left hand radiographs at each visit. These maturity status indicators are used to determine whether a patient is ready for brace weaning.

3. Scoliosis specific parameters

All AIS patients at DKCH will have standing PA and lateral whole spine radiographs taken at each follow-up visit with the EOS® (biplanar x-ray system); from which, the curve type (i.e. double primary, thoracic, thoracolumbar, and lumbar, according to the SRS Spinal Classification Nomenclature), number of curves, coronal Cobb angle, and apex will be assessed. Additional data regarding balance parameters include clinical rib hump (apical trunk rotation) measurement by scoliometer (measured routinely by a designated clinic nurse), radiological measurements of the T1 tilt, shoulder height, truncal shift, listing (C7-central sacral vertebral line deviation), sagittal vertical axis, lumbar lordosis (L1-S1) and thoracic kyphosis (T5-12). For all radiographic parameters, the two independent investigators will perform all measurements independently. When the difference in measurements are less than 5 degrees or 5mm, the mean of the two measurements will be taken and reported on the source document. When the discrepancy is more than 5 degrees or 5mm, the two assessors will meet and discuss, and come up with a joint concluded value for reporting.

4. Mode of treatment

Data to be retrieved include the duration of brace treatment, as well as the corresponding start and end dates. Any previous complications from bracing including curve deterioration, abrasions and bruising will be recorded.

5. Brace compliance and routine measures for the gradual bracing group

All subjects undergoing gradual brace weaning will have thermal sensors to monitor

compliance to our weaning protocol. An additional orthotic assessment will be performed as usual prior to clinic consultation to scan the sensor for compliance data. To standardize the taught physical activity between the two groups, no additional physiotherapy sessions will be arranged for scoliosis specific exercises.

6. Health-related quality of life and utility score assessments

A. The refined Chinese version of the Scoliosis Research Society-22 (SRS-22r) questionnaire

The SRS-22r is a 22-item quality of life questionnaire for patients with spinal deformity. It comprises of 5 scales measuring function/activity, pain, self-image/appearance, mental health, and satisfaction with management. The investigators have already translated and validated the Chinese version of the questionnaire.[25] The SRS-22r has been routinely administered to all patients with scoliosis seen at DKCH through a web-enabled system. Moreover, multiple data-points should be available, as patients are required to fill in a questionnaire on every visit to the clinic.

B. The EuroQol-5-dimension (EQ-5D) utility score

The EQ-5D comprises of 5 questions that provides the health utility input of QALYs for cost-utility of interventions. This measure has grown popularity and has been used for health-related quality of life and utility score assessment. EQ-5D scores are valid, reliable and sensitive for utility assessment in AIS patients. We will utilize the EQ-5D-5L for this study.

C. EuroQol-visual analogue scale (EQ-VAS)

EQ-VAS ranges from 1 to 100, which represents the patient’s own perception of his/her own health status on the day of clinical visit.

Summary table of assessments can be seen in Table 1.

<u>Table 1. Summary Table of assessments</u>

Type of parameters	Study Parameters
Socio-demographics	Date of birth, gender, ethnicity, school grade number of siblings/family history of scoliosis
Maturity parameters	Clinical<br>Standing and sitting height, arm span, date of menarche<br>Radiological<br>Risser sign, distal radius and ulna (DRU) classification, Risser staging
Scoliosis specific parameters	Coronal Cobb angle<br>Truncal balance (truncal shift, C7-central sacral vertical line deviation)<br>Curve type and number of curves, apex location, apical trunk rotation<br>Sagittal vertical axis (SVA)<br>L1-S1 lumbar lordosis<br>T5-12 thoracic kyphosis
Mode of treatment	Duration of brace treatment<br>Complications of bracing
Brace compliance and routine measures	Compliance (hours) by thermal sensor
Health-related quality of life (HRQoL) measures	Refined Scoliosis Research Society-22 (SRS-22r) questionnaire<br>EuroQol-5 dimension (EQ-5D) utility score<br>EuroQol-visual analogue scale (EQ-VAS).
Safety assessment	Adverse event

7. BLINDED OUTCOME ASSESSMENT BY INDEPENDENT INVESTIGATORS

All scoliosis specific parameters will be evaluated by two independent investigators separately. These are radiographs which will be anonymized during assessment, and scoliometer measurements done by a designated nurse who is not involved in this study. Investigators will be blinded to the study treatment throughout the study period.

All statistical analyses will be performed using SPSS Statistics v. 28.0. (Armonk, NY, IBM Corp). Further details of statistical method and analysis will be described in a statistical analysis plan.

8. Safety Assessment

All adverse events (AE) will be collected in this study. The description of AE, start date and end date, severity, relationship to the study protocol and outcome of the event, will be recorded. Any AE encountered will be minimal and are isolated to complications of brace-wear for the gradual weaning group. These include abrasions and bruising.

9. Ethics and Regulation

The study will be conducted in accordance with the protocol, ICH GCP E6 (R2), ethical principles that have their origin in the Declaration of Helsinki and all applicable regulations. All serious violation of the above-mentioned standards/regulations must be reported by principal investigator to Independent Ethics Committee (IEC)/Institutional Review Board (IRB), and local regulatory authority when applicable. A serious violation is a breach of the conditions and principles of ICH GCP E6 (R2), study protocol and applicable regulations, which is likely to affect patient protection and data integrity of the study.

11. Informed Consent Form

The Informed Consent Form (ICF) used in the study must be reviewed and approved by the local IEC/IRB. Prior to any study-related screening procedures being performed on the patients, the Investigator or his/her designees should explain all aspects of the study to the patients and answer all their questions about this study.

The Investigator should provide the patient ample time and opportunity to inquire about details of the study and to decide whether or not to participate in the trial. If the patient voluntarily agrees to participate in the study, the written ICF should be signed and personally dated by the patient. The Investigator who conducts the informed consent discussion should also personally sign and date on the ICF.

A copy of the signed ICF will be given to the patient and the original will be placed in the Investigator’s File. The Investigator should document on the source document the date of obtaining the signed consent and that the patient received a signed copy.

12. Data Safety and Monitoring

Principal Investigator shall protect the confidentiality of patients. Personal data is defined as any information or data that relates to an identified or identifiable patients or different pieces of information, which collect together can lead to the identification of a patient. Personal data includes but not limited to patient’s medical records and laboratory results.

By obtaining patient’s written consent, the Investigator has the patient’s permission and shall permit the regulatory authorities, IRB/IEC representatives to get direct access to patient's personal data for data verification purposes.

Study data should not contain any patient’s identification. All persons involved in the study are bound by this confidentiality clause. PI may only be disclosed the study data to third-party collaborator(s) as authorised by the ICF signed by the patients.

13. Potential Benefits

Scoliosis is a lateral curvature of the spine, affecting every population worldwide. It is the most common spinal deformity that affects children and its most common form is AIS. Curve progression occurs with growth and thus timely intervention with bracing is important to control the deformity. However, prolonged bracing may lead to osteoporosis, poor muscle endurance and reduced spine mobility. Brace weaning is initiated when patients are skeletally mature. However, current brace weaning protocols are not standardized and are usually clinician dependent. Some clinicians may suggest a gradual brace weaning protocol in hope of the spine viscoelastic properties to allow maintenance of in-brace curve characteristics while some may just terminate brace treatment completely without any weaning process. Decisions are thus variable and not based on any evidence.

By determining the best brace weaning protocol associated with optimum outcomes of maintaining Cobb angle and truncal balance, we can provide our patients with evidence-based treatment decisions that produce the greatest benefit. There are increasing demands for high-quality healthcare in including designing evidence-based management protocols, especially for the commonest pediatric spinal deformity that is AIS. There is potential for our results to support a standardized universal brace weaning protocol. We can provide good evidence-based care for tens of thousands of AIS children around the world. The results are impactful not only local but also on a global scale.

14. Reference

1. Weinstein SL, Dolan LA, Spratt KF, Peterson KK, Spoonamore MJ, Ponseti IV. Health and function of patients with untreated idiopathic scoliosis: a 50-year natural history study. JAMA. 2003;289(5):559-67. PubMed PMID: 12578488.

2. Reamy BV, Slakey JB. Adolescent idiopathic scoliosis: review and current concepts. Am Fam Physician. 2001;64(1):111-6. PubMed PMID: 11456428.

3. Fong DY, Cheung KM, Wong YW, Wan YY, Lee CF, Lam TP, et al. A population-based cohort study of 394,401 children followed for 10 years exhibits sustained effectiveness of scoliosis screening. The spine journal : official journal of the North American Spine Society. 2015;15(5):825-33. doi: 10.1016/j.spinee.2015.01.019. PubMed PMID: 25615844.

4. Lee CF, Fong DY, Cheung KM, Cheng JC, Ng BK, Lam TP, et al. Referral criteria for school scoliosis screening: assessment and recommendations based on a large longitudinally followed cohort. Spine (Phila Pa 1976). 2010;35(25):E1492-8. doi: 10.1097/BRS.0b013e3181ecf3fe. PubMed PMID: 21102278.

5. Lonstein JE, Winter RB, Bradford DS, Ogilvie JW. Moe's textbook of scoliosis and other spinal deformities. 3rd ed. Philadelphia: W.B. Saunders; 1995.

6. Weinstein SL, Ponseti IV. Curve progression in idiopathic scoliosis. The Journal of bone and joint surgery American volume. 1983;65(4):447-55. PubMed PMID: 6833318.

7. Rahman T, Bowen JR, Takemitsu M, Scott C. The association between brace compliance and outcome for patients with idiopathic scoliosis. J Pediatr Orthop. 2005;25(4):420-2. PubMed PMID: 15958887.

8. Fong DY, Cheung KM, Wong YW, Cheung WY, Fu IC, Kuong EE, et al. An alternative to a randomised control design for assessing the efficacy and effectiveness of bracing in adolescent idiopathic scoliosis. Bone Joint J. 2015;97-B(7):973-81. doi: 10.1302/0301-620X.97B7.35147. PubMed PMID: 26130355.

9. Weinstein SL, Dolan LA, Wright JG, Dobbs MB. Effects of bracing in adolescents with idiopathic scoliosis. N Engl J Med. 2013;369(16):1512-21. doi: 10.1056/NEJMoa1307337. PubMed PMID: 24047455; PubMed Central PMCID: PMCPMC3913566.

10. Maruyama T, Grivas TB, Kaspiris A. Effectiveness and outcomes of brace treatment: a systematic review. Physiother Theory Pract. 2011;27(1):26-42. doi: 10.3109/09593985.2010.503989. PubMed PMID: 21198404.

11. Katz DE, Durrani AA. Factors that influence outcome in bracing large curves in patients with adolescent idiopathic scoliosis. Spine (Phila Pa 1976). 2001;26(21):2354-61. PubMed PMID: 11679821.

12. Landauer F, Wimmer C, Behensky H. Estimating the final outcome of brace treatment for idiopathic thoracic scoliosis at 6-month follow-up. Pediatr Rehabil. 2003;6(3-4):201-7. doi: 10.1080/13638490310001636817. PubMed PMID: 14713586.

13. Cheung KM, Cheng EY, Chan SC, Yeung KW, Luk KD. Outcome assessment of bracing in adolescent idiopathic scoliosis by the use of the SRS-22 questionnaire. Int Orthop. 2007;31(4):507-11. doi: 10.1007/s00264-006-0209-5. PubMed PMID: 16896864; PubMed Central PMCID: PMC2267629.

14. Climent JM, Sanchez J. Impact of the type of brace on the quality of life of Adolescents with Spine Deformities. Spine (Phila Pa 1976). 1999;24(18):1903-8. PubMed PMID: 10515014.

15. Noonan KJ, Dolan LA, Jacobson WC, Weinstein SL. Long-term psychosocial characteristics of patients treated for idiopathic scoliosis. J Pediatr Orthop. 1997;17(6):712-7. PubMed PMID: 9591971.

16. Odermatt D, Mathieu PA, Beausejour M, Labelle H, Aubin CE. Electromyography of scoliotic patients treated with a brace. J Orthop Res. 2003;21(5):931-6. doi: 10.1016/S0736-0266(03)00038-X. PubMed PMID: 12919883.

17. Ugwonali OF, Lomas G, Choe JC, Hyman JE, Lee FY, Vitale MG, et al. Effect of bracing on the quality of life of adolescents with idiopathic scoliosis. Spine J. 2004;4(3):254-60. doi: 10.1016/j.spinee.2003.12.001. PubMed PMID: 15125845.

18. Vasiliadis E, Grivas TB, Savvidou O, Triantafyllopoulos G. The influence of brace on quality of life of adolescents with idiopathic scoliosis. Stud Health Technol Inform. 2006;123:352-6. PubMed PMID: 17108451.

19. Li XF, Li H, Liu ZD, Dai LY. Low bone mineral status in adolescent idiopathic scoliosis. Eur Spine J. 2008;17(11):1431-40. doi: 10.1007/s00586-008-0757-z. PubMed PMID: 18751741; PubMed Central PMCID: PMCPMC2583185.

20. Rogala EJ, Drummond DS, Gurr J. Scoliosis: incidence and natural history. A prospective epidemiological study. J Bone Joint Surg Am. 1978;60(2):173-6. PubMed PMID: 641080.

21. Danielsson AJ, Romberg K, Nachemson AL. Spinal range of motion, muscle endurance, and back pain and function at least 20 years after fusion or brace treatment for adolescent idiopathic scoliosis: a case-control study. Spine (Phila Pa 1976). 2006;31(3):275-83. doi: 10.1097/01.brs.0000197652.52890.71. PubMed PMID: 16449899.

22. Cheung JP, Cheung PW, Samartzis D, Cheung KM, Luk KD. The use of the distal radius and ulna classification for the prediction of growth: peak growth spurt and growth cessation. Bone Joint J. 2016;98-B(12):1689-96. doi: 10.1302/0301-620X.98B12.BJJ-2016-0158.R1. PubMed PMID: 27909133.

23. Li M, Wong MS, Luk KD, Wong KW, Cheung KM. Time-dependent response of

scoliotic curvature to orthotic intervention: when should a radiograph be obtained after putting on or taking off a spinal orthosis? Spine (Phila Pa 1976). 2014;39(17):1408-16. doi: 10.1097/BRS.0000000000000423. PubMed PMID: 24859575.

24. Shi B, Guo J, Mao S, Wang Z, Yu FW, Lee KM, et al. Curve Progression in Adolescent Idiopathic Scoliosis With a Minimum of 2 Years' Follow-up After Completed Brace Weaning With Reference to the SRS Standardized Criteria. Spine Deform. 2016;4(3):200-5. doi: 10.1016/j.jspd.2015.12.002. PubMed PMID: 27927503.

25. Cheung KM, Senkoylu A, Alanay A, Genc Y, Lau S, Luk KD. Reliability and concurrent validity of the adapted Chinese version of Scoliosis Research Society-22 (SRS-22) questionnaire. Spine. 2007;32(10):1141-5. Epub 2007/05/02. doi: 10.1097/01.brs.0000261562.48888.e3. PubMed PMID: 17471100.

STUDY PROTOCOL

A randomized controlled trial comparing gradual and immediate brace weaning for clinical management of adolescent idiopathic scoliosis patients

Protocol Number and Version

Version 2.0 dated 10 Dec 2018

(Additions are highlighted in light gray)

Funding

Health and Medical Research Fund

1. INTRODUCTION

Scoliosi is a 3-dimensional deformity characterized as a lateral curvature of the spine in the coronal plane as measured by Cobb angle >10 degrees on plain radiographs. Patients with adolescent idiopathic scoliosis (AIS) may develop curve progression if left untreated, which can result in back pain, cosmetic disfigurement and early death from cardiopulmonary compromise.[1] The most common form of scoliosis is idiopathic and is usually detected during adolescence (10-18 years of age).[2] Based on the investigators' large-scale and internationally renowned Hong Kong-based scoliosis school screening program [3, 4], the prevalence of spine curvatures were as follows: ≥10 degrees was 2.2% for boys and 4.8% for girls; ≥20 degrees was 0.7% for boys and 2.8% for girls; and for ≥40 degrees the prevalence was 0.1% for boys and 0.4% for girls.[3] It is important to note that approximately only 78% of students in Hong Kong have participated in the school screening system. Hence, the prevalence may perhaps be an underestimation. Currently, in Hong Kong, all children diagnosed with AIS and noted to have a ≥20 degrees curve are referred to one of two scoliosis clinics for management as those who are skeletally immature have potential to deteriorate during growth and may be treated by bracing.[5] To place into context the magnitude of service required, "450-500 new braces" are prescribed per year in our clinic alone.

Bracing is the most common treatment method for controlling AIS progression and its main purpose is to prevent curves from reaching the surgical threshold of >50 degrees. However, prevention of any Cobb angle progression is also beneficial. Normally, at the end of skeletal growth, there is a significant reduction in risk of further curve progression. However, there is an increased risk of continued progression into adulthood of at least 1 degree per year for curvatures of ≥40 degrees at skeletal maturity. Hence, not only is a smaller Cobb angle at skeletal maturity more cosmetically acceptable, preventing the 40 degree threshold can reduce the risk of adulthood deterioration and surgery as suggested by natural history studies.[1, 6] Good brace compliance with more than 20 hours of daily use is proven to be effective in reducing curve progression.[7] High-level and established evidence have been published which support its efficacy.[8-10] Specifically, Weinstein et al[9] have demonstrated in a multicenter study that in patients with high-risk curves bracing significantly decreased curve progression and the need for surgery. Based on that study,

longer brace wearing decreased curve progression and the need for surgery.

At the end of skeletal growth, treatment is completed and braces are removed or "weaned".[7, 11, 12] This decision should be made in a timely fashion as drawbacks and complications have been noted with prolonged brace use. Prolonged bracing in children may reduce spinal mobility, lead to poor body image and self-esteem, and worsen quality of life.[13-18] There is a risk of osteoporosis and the prevalence of low bone mineral density in AIS patients may involve up to 38% of individuals.[19] A major reason why osteoporosis may occur is the reduced physical activity and use of core musculature due to brace immobilization. Although bracing improves trunk posture, it limits active body movement.[20] In the long-term, reduced spinal mobility and muscle endurance may occur up to 20 years after bracing has completed.[21] We currently have accurate parameters that help determine when patients reach skeletal maturity and no longer require further bracing.[22] However, **the manner in which brace treatment should be weaned is "clinician-dependent" and without any supporting evidence.**

For brace weaning, AIS patients can only rely on the surgeon's decision rather than evidence-based guidelines. This may be a **slow weaning process**, whereby the duration of brace wear is slowly reduced or changed to night-use only, or may be a **rapid immediate removal** of the brace. The main rationale for a slow gradual weaning process over a period of at least 6 months is to allow the spine to adapt to an environment without loading by the brace while maintaining the corrective posture. We postulate that by this method, the curve magnitude measured by the Cobb angle and the overall truncal balance and posture may be maintained even after brace removal. The resulting curve magnitude is the commonly used outcome measure of treatment as it has bearing on the likelihood of curve deterioration and surgery. However, truncal balance may be a larger concern for patients due to its larger impact on overall cosmesis. Whether this theory of gradual weaning benefits holds true is unknown but is supported by our knowledge of the spine's viscoelastic properties. The viscoelasticity of spinal structures including the ligaments, joint capsules and intervertebral discs allows deformation changes while loading and unloading the spine.[23] A lag-time response of spinal soft tissues to these changes have been demonstrated. Despite our limited knowledge on the effects of gradual weaning on the spine, deterioration in Cobb angle and truncal balance parameters may lead to worse cosmesis and quality of life, and possible need for surgery. Hence, **lack of an optimal brace weaning protocol is a significant**

limitation in our current management of AIS patients.

In this modern era, there is increasing emphasis placed on evidence-based health-care. Clinicians and health-care administrators are tasked to provide the best care for our patients. While some clinicians may terminate brace treatment with a gradual weaning period, others are content with immediate brace removal to avoid prolonged brace treatment. Due to the lack of evidence for either method, it is an appropriate time to determine any advantages of gradual brace weaning for AIS patients. Bracing is a commonly used tool for preventing curve progression, and it is imperative to deliver the best treatment protocol to these children. With this purpose, we propose a randomized controlled trial to determine whether gradual weaning does benefit over immediate weaning.

2. STUDY OBJECTIVES

a. Primary Aim

To compare the degree of Cobb angle maintenance between immediate removal and gradual brace weaning protocols for AIS patients at 1 year after brace weaning.

Our hypothesis is that gradual brace weaning allows better adaptation to an unloaded environment without the brace in AIS patients, thereby resulting in better maintenance of the Cobb angle at 1 year after brace weaning.

b. Secondary Aim

i. To compare the degree of truncal balance maintenance between immediate removal and gradual brace weaning protocols for AIS patients after brace weaning.

We hypothesize that AIS patients with gradual brace weaning allows better adaptation to an unloaded environment without the brace thereby resulting with better maintenance of

truncal balance.

ii. To determine any differences in health-related quality of life (HRQOL) between immediate removal and gradual weaning protocols.

We hypothesize that due to improved Cobb angle and truncal balance maintenance, the HRQOL is better for the gradual weaning protocol.

3. STUDY DESIGN

a. Overall Study Design

This is an open-labelled, randomized controlled trial for testing two brace weaning protocols, namely immediate removal of brace to that of gradual brace weaning over a course of 6 months and followed-up for 12 months and 24 months. The Clinical Trials Centre (CTC) at the University of Hong Kong is involved in reviewing the study design, statistical methodology of the concept, sample size calculation and will also aid in data and study site monitoring after study commencement.

b. Definition of Study Intervention

As there is no definition for “brace weaning”, we adopt the protocol that our center has been using which has also been previously published.[24] Hence, we will be comparing two groups:

1) gradual brace weaning with the brace wearing time shortened to night wearing for 6 more months before stopping bracing altogether or

2) immediate brace weaning on the day of patient recruitment.

c. Study Duration

All subjects will be followed-up longitudinally the primary end point at 1 year after brace weaning has been issued. A final assessment time point at 24 months is added to assess the long term efficacy [25] of weaning protocol in the maintenance of curve magnitude and truncal balance. Therefore, this includes three clinic visits with one at 6 months to complete the gradual brace weaning protocol, another at 1 year and a final visit at 2 years to observe for any delayed changes to the overall curvature and balance, including loss of truncal balance and Cobb angle deterioration. This follow-up duration is based on previous work suggesting that the highest risk of post-brace weaning curve progression occurs within 6 months of stopping brace wear.[24] A 1-year collection of data is adequate for seeing differences between the two groups. This does not deviate significantly from our usual care as all AIS subjects in our clinic are followed-up at 6-monthly intervals. The 2 year visit will offer additional information about the effects of brace weaning protocols in the long term.

4. STUDY POPULATION

We will recruit AIS patients managed at the Duchess of Kent Children's Hospital (DKCH) in Hong Kong. DKCH is one of two only scoliosis centers in Hong Kong that manages AIS. Being under the same umbrella of the Hong Kong healthcare system, the data gathered at DKCH is representative of the whole of Hong Kong with regards to the same referral, treatment protocol and healthcare cost system.

c. Inclusion Criteria

A patient will be eligible for study participation if he/she meets all the following criteria:

- All AIS patients treated with underarm (Boston) bracing who are ready to undergo brace weaning
- Primary curve ranging between 20 and 40 degrees of Cobb angle measurement

- Have reached skeletal maturity based on the Scoliosis Research Society (SRS) standardized criteria: Risser stage ≥4, >2 years post-menarche, and no growth between 2 visits.

d. Exclusion Criteria

A patient will be excluded from the study if he/she meets any of the following criteria:

- Non-AIS patients
- Those undergoing Milwaukee bracing
- Patients who undergo brace weaning not because of skeletal maturity but because of the need for surgical intervention
- Cognitively impaired, unable to comply with study follow-up and protocol issued
- Refused consent for study.

5. RANDOMIAZATION AND ALLOCATION

Block randomization with randomly selected even number of block sizes will be used to ensure a balance in sample size between the study groups. A master randomization list will be generated by a statistician a priori. A web-based randomization application will be set up and the randomization list will be uploaded. Using the application, eligible patients who have consented to take part in the study will be randomly allocated to one of the two protocols by the project coordinator. The master randomization list will be managed independently by the project coordinator and concealed to all research staff until the study database closure. Attending specialist will perform patient recruitment and informed consent procedure. Project coordinator will be responsible for allocating recruited patients to either of the two arm of the trial.

6. STUDY ASSESSMENTS

All subjects will undergo weight, standing and sitting height, and arm span measurements, HRQOL and utility measurements, and whole spine standing posteroanterior (PA) and lateral radiographs at each clinic visit. The refined Scoliosis Research Society 22-item (SRS-22r) questionnaire and the EuroQoL 5-Dimension 5-Level (EQ5D) questionnaire will thus be provided at every visit for HRQOL and utility assessment. For all patients undergoing bracing, an orthotist assessment is also performed at each clinic visit for any brace modification and education. Further details regarding the measurements assessed are listed in the following pages.

Study Outcomes

a. Primary outcome

- Change in Cobb angle from baseline to 6 months, 1 year and 2 years follow-up.

b. Secondary outcomes

- Change in truncal balance from baseline to 6 months, 1 year and 2 years follow-up.
- SRS-22r total score at 6 months, 1 year and 2 years follow-up.
- EQ-5D utility score at 6 months, 1 year and 2-years follow-up.

Study Measurements Assessed

1. Socio-demographics

These data include the date of birth, gender, ethnic group, school grade, the number of siblings and siblings also under scoliosis management.

2. Body size and maturity parameters

This includes standing and sitting height, weight, and arm span at each follow-up

visit. All of these clinical parameters are measured routinely and hence, the height change since the previous follow-up can be measured. For girls, the menarche status and the start date of menarche will also be extracted. The Risser sign is determined by evaluating the extent of ossification of the iliac apophysis on the same PA spine radiographs used to assess the spine radiographic measurements. The bone age by the distal radius and ulna classification [22] will also be measured on left hand radiographs at each visit. These maturity status indicators are used to determine whether a patient is ready for brace weaning.

3. Scoliosis specific parameters

All AIS patients at DKCH will have standing PA and lateral whole spine radiographs taken at each follow-up visit with the EOS® (biplanar x-ray system); from which, the curve type (i.e. double primary, thoracic, thoracolumbar, and lumbar, according to the SRS Spinal Classification Nomenclature), number of curves, coronal Cobb angle, and apex will be assessed. Additional data regarding balance parameters include clinical rib hump (apical trunk rotation) measurement by scoliometer (measured routinely by a designated clinic nurse), radiological measurements of the T1 tilt, shoulder height, truncal shift, listing (C7-central sacral vertebral line deviation), sagittal vertical axis, lumbar lordosis (L1-S1) and thoracic kyphosis (T5-12). For all radiographic parameters, the two independent investigators will perform all measurements independently. When the difference in measurements are less than 5 degrees or 5mm, the mean of the two measurements will be taken and reported on the source document. When the discrepancy is more than 5 degrees or 5mm, the two assessors will meet and discuss, and come up with a joint concluded value for reporting.

4. Mode of treatment

Data to be retrieved include the duration of brace treatment, as well as the corresponding start and end dates. Any previous complications from bracing including curve deterioration, abrasions and bruising will be recorded.

5. Brace compliance and routine measures for the gradual bracing group

All subjects undergoing gradual brace weaning will have thermal sensors to monitor

compliance to our weaning protocol. An additional orthotic assessment will be performed as usual prior to clinic consultation to scan the sensor for compliance data. This will be considered in any cost-utility assessment. To standardize the taught physical activity between the two groups, no additional physiotherapy sessions will be arranged for scoliosis specific exercises.

6. Health-related quality of life and utility score assessments

A. The refined Chinese version of the Scoliosis Research Society-22 (SRS-22r) questionnaire

The SRS-22r is a 22-item quality of life questionnaire for patients with spinal deformity. It comprises of 5 scales measuring function/activity, pain, self-image/appearance, mental health, and satisfaction with management. The investigators have already translated and validated the Chinese version of the questionnaire.[26] The SRS-22r has been routinely administered to all patients with scoliosis seen at DKCH through a web-enabled system. Moreover, multiple data-points should be available, as patients are required to fill in a questionnaire on every visit to the clinic.

B. The EuroQol-5-dimension (EQ-5D) utility score

The EQ-5D comprises of 5 questions that provides the health utility input of QALYs for cost-utility of interventions. This measure has grown popularity and has been used for health-related quality of life and utility score assessment. EQ-5D scores are valid, reliable and sensitive for utility assessment in AIS patients. We will utilize the EQ-5D-5L for this study.

C. EuroQol-visual analogue scale (EQ-VAS)

EQ-VAS ranges from 1 to 100, which represents the patient’s own perception of his/her own health status on the day of clinical visit.

Summary table of assessments can be seen in Table 1.

Table 1. Summary Table of assessments

**Type of parameters**	**Study Parameters**
Socio-demographics	Date of birth, gender, ethnicity, school grade number of siblings/family history of scoliosis
Maturity parameters	Clinical<br>Standing and sitting height, arm span, date of menarche<br>Radiological<br>Risser sign, distal radius and ulna (DRU) classification, Risser staging
Scoliosis specific parameters	Coronal Cobb angle<br>Truncal balance (truncal shift, C7-central sacral vertical line deviation)<br>Curve type and number of curves, apex location, apical trunk rotation<br>Sagittal vertical axis (SVA)<br>L1-S1 lumbar lordosis<br>T5-12 thoracic kyphosis
Mode of treatment	Duration of brace treatment<br>Complications of bracing
Brace compliance and routine measures	Compliance (hours) by thermal sensor
Health-related quality of life (HRQoL) measures	Refined Scoliosis Research Society-22 (SRS-22r) questionnaire<br>EuroQol-5 dimension (EQ-5D) utility score<br>EuroQol-visual analogue scale (EQ-VAS).
Safety assessment	Adverse event

7. Blinded Outcome Assessment by Independent Investigators

All scoliosis specific parameters will be evaluated by two independent investigators separately. These are radiographs which will be anonymized during assessment, and scoliometer measurements done by a designated nurse who is not involved in this study. Investigators will be blinded to the study treatment throughout the study period.

All statistical analyses will be performed using SPSS Statistics v. 28.0. (Armonk, NY, IBM Corp). Further details of statistical method and analysis will be described in a statistical analysis plan.

8. Safety Assessment

All adverse events (AE) will be collected in this study. The description of AE, start date and end date, severity, relationship to the study protocol and outcome of the event, will be recorded. Any AE encountered will be minimal and are isolated to complications of brace-wear for the gradual weaning group. These include abrasions and bruising.

9. Ethics and Regulation

The study will be conducted in accordance with the protocol, ICH GCP E6 (R2), ethical principles that have their origin in the Declaration of Helsinki and all applicable regulations. All serious violation of the above-mentioned standards/regulations must be reported by principal investigator to Independent Ethics Committee (IEC)/Institutional Review Board (IRB), and local regulatory authority when applicable. A serious violation is a breach of the conditions and principles of ICH GCP E6 (R2), study protocol and applicable regulations, which is likely to affect patient protection and data integrity of the study.

11. Informed Consent Form

The Informed Consent Form (ICF) used in the study must be reviewed and approved by the local IEC/IRB. Prior to any study-related screening procedures being performed on the patients, the Investigator or his/her designees should explain all aspects of the study to the patients and answer all their questions about this study.

The Investigator should provide the patient ample time and opportunity to inquire about details of the study and to decide whether or not to participate in the trial. If the patient voluntarily agrees to participate in the study, the written ICF should be signed and personally dated by the patient. The Investigator who conducts the informed consent discussion should also personally sign and date on the ICF.

A copy of the signed ICF will be given to the patient and the original will be placed

in the Investigator’s File. The Investigator should document on the source document the date of obtaining the signed consent and that the patient received a signed copy.

12. DATA SAFETY AND MONITORING

Principal Investigator shall protect the confidentiality of patients. Personal data is defined as any information or data that relates to an identified or identifiable patients or different pieces of information, which collect together can lead to the identification of a patient. Personal data includes but not limited to patient’s medical records and laboratory results.

By obtaining patient’s written consent, the Investigator has the patient’s permission and shall permit the regulatory authorities, IRB/IEC representatives to get direct access to patient's personal data for data verification purposes.

Study data should not contain any patient’s identification. All persons involved in the study are bound by this confidentiality clause. PI may only be disclosed the study data to third-party collaborator(s) as authorised by the ICF signed by the patients.

13. POTENTIAL BENEFITS

Scoliosis is a lateral curvature of the spine, affecting every population worldwide. It is the most common spinal deformity that affects children and its most common form is AIS. Curve progression occurs with growth and thus timely intervention with bracing is important to control the deformity. However, prolonged bracing may lead to osteoporosis, poor muscle endurance and reduced spine mobility. Brace weaning is initiated when patients are skeletally mature. However, current brace weaning protocols are not standardized and are usually clinician dependent. Some clinicians may suggest a gradual brace weaning protocol in hope of the spine viscoelastic properties to allow maintenance of in-brace curve characteristics while some may just terminate brace treatment completely without any weaning process. Decisions are thus variable and not based on any evidence.

By determining the best brace weaning protocol associated with optimum outcomes

of maintaining Cobb angle and truncal balance, we can provide our patients with evidence-based treatment decisions that produce the greatest benefit. There are increasing demands for high-quality healthcare in including designing evidence-based management protocols, especially for the commonest pediatric spinal deformity that is AIS. There is potential for our results to support a standardized universal brace weaning protocol. We can provide good evidence-based care for tens of thousands of AIS children around the world. The results are impactful not only local but also on a global scale.

14. REFERENCE

1. Weinstein SL, Dolan LA, Spratt KF, Peterson KK, Spoonamore MJ, Ponseti IV. Health and function of patients with untreated idiopathic scoliosis: a 50-year natural history study. JAMA. 2003;289(5):559-67. PubMed PMID: 12578488.

2. Reamy BV, Slakey JB. Adolescent idiopathic scoliosis: review and current concepts. Am Fam Physician. 2001;64(1):111-6. PubMed PMID: 11456428.

3. Fong DY, Cheung KM, Wong YW, Wan YY, Lee CF, Lam TP, et al. A population-based cohort study of 394,401 children followed for 10 years exhibits sustained effectiveness of scoliosis screening. The spine journal : official journal of the North American Spine Society. 2015;15(5):825-33. doi: 10.1016/j.spinee.2015.01.019. PubMed PMID: 25615844.

4. Lee CF, Fong DY, Cheung KM, Cheng JC, Ng BK, Lam TP, et al. Referral criteria for school scoliosis screening: assessment and recommendations based on a large longitudinally followed cohort. Spine (Phila Pa 1976). 2010;35(25):E1492-8. doi: 10.1097/BRS.0b013e3181ecf3fe. PubMed PMID: 21102278.

5. Lonstein JE, Winter RB, Bradford DS, Ogilvie JW. Moe's textbook of scoliosis and other spinal deformities. 3rd ed. Philadelphia: W.B. Saunders; 1995.

6. Weinstein SL, Ponseti IV. Curve progression in idiopathic scoliosis. The Journal of bone and joint surgery American volume. 1983;65(4):447-55. PubMed PMID: 6833318.

7. Rahman T, Bowen JR, Takemitsu M, Scott C. The association between brace

compliance and outcome for patients with idiopathic scoliosis. J Pediatr Orthop. 2005;25(4):420-2. PubMed PMID: 15958887.

8. Fong DY, Cheung KM, Wong YW, Cheung WY, Fu IC, Kuong EE, et al. An alternative to a randomised control design for assessing the efficacy and effectiveness of bracing in adolescent idiopathic scoliosis. Bone Joint J. 2015;97-B(7):973-81. doi: 10.1302/0301-620X.97B7.35147. PubMed PMID: 26130355.

9. Weinstein SL, Dolan LA, Wright JG, Dobbs MB. Effects of bracing in adolescents with idiopathic scoliosis. N Engl J Med. 2013;369(16):1512-21. doi: 10.1056/NEJMoa1307337. PubMed PMID: 24047455; PubMed Central PMCID: PMCPMC3913566.

10. Maruyama T, Grivas TB, Kaspiris A. Effectiveness and outcomes of brace treatment: a systematic review. Physiother Theory Pract. 2011;27(1):26-42. doi: 10.3109/09593985.2010.503989. PubMed PMID: 21198404.

11. Katz DE, Durrani AA. Factors that influence outcome in bracing large curves in patients with adolescent idiopathic scoliosis. Spine (Phila Pa 1976). 2001;26(21):2354-61. PubMed PMID: 11679821.

12. Landauer F, Wimmer C, Behensky H. Estimating the final outcome of brace treatment for idiopathic thoracic scoliosis at 6-month follow-up. Pediatr Rehabil. 2003;6(3-4):201-7. doi: 10.1080/13638490310001636817. PubMed PMID: 14713586.

13. Cheung KM, Cheng EY, Chan SC, Yeung KW, Luk KD. Outcome assessment of bracing in adolescent idiopathic scoliosis by the use of the SRS-22 questionnaire. Int Orthop. 2007;31(4):507-11. doi: 10.1007/s00264-006-0209-5. PubMed PMID: 16896864; PubMed Central PMCID: PMC2267629.

14. Climent JM, Sanchez J. Impact of the type of brace on the quality of life of Adolescents with Spine Deformities. Spine (Phila Pa 1976). 1999;24(18):1903-8. PubMed PMID: 10515014.

15. Noonan KJ, Dolan LA, Jacobson WC, Weinstein SL. Long-term psychosocial

characteristics of patients treated for idiopathic scoliosis. J Pediatr Orthop. 1997;17(6):712-7. PubMed PMID: 9591971.

16. Odermatt D, Mathieu PA, Beausejour M, Labelle H, Aubin CE. Electromyography of scoliotic patients treated with a brace. J Orthop Res. 2003;21(5):931-6. doi: 10.1016/S0736-0266(03)00038-X. PubMed PMID: 12919883.

17. Ugwonali OF, Lomas G, Choe JC, Hyman JE, Lee FY, Vitale MG, et al. Effect of bracing on the quality of life of adolescents with idiopathic scoliosis. Spine J. 2004;4(3):254-60. doi: 10.1016/j.spinee.2003.12.001. PubMed PMID: 15125845.

18. Vasiliadis E, Grivas TB, Savvidou O, Triantafyllopoulos G. The influence of brace on quality of life of adolescents with idiopathic scoliosis. Stud Health Technol Inform. 2006;123:352-6. PubMed PMID: 17108451.

19. Li XF, Li H, Liu ZD, Dai LY. Low bone mineral status in adolescent idiopathic scoliosis. Eur Spine J. 2008;17(11):1431-40. doi: 10.1007/s00586-008-0757-z. PubMed PMID: 18751741; PubMed Central PMCID: PMCPMC2583185.

20. Rogala EJ, Drummond DS, Gurr J. Scoliosis: incidence and natural history. A prospective epidemiological study. J Bone Joint Surg Am. 1978;60(2):173-6. PubMed PMID: 641080.

21. Danielsson AJ, Romberg K, Nachemson AL. Spinal range of motion, muscle endurance, and back pain and function at least 20 years after fusion or brace treatment for adolescent idiopathic scoliosis: a case-control study. Spine (Phila Pa 1976). 2006;31(3):275-83. doi: 10.1097/01.brs.0000197652.52890.71. PubMed PMID: 16449899.

22. Cheung JP, Cheung PW, Samartzis D, Cheung KM, Luk KD. The use of the distal radius and ulna classification for the prediction of growth: peak growth spurt and growth cessation. Bone Joint J. 2016;98-B(12):1689-96. doi: 10.1302/0301-620X.98B12.BJJ-2016-0158.R1. PubMed PMID: 27909133.

23. Li M, Wong MS, Luk KD, Wong KW, Cheung KM. Time-dependent response of scoliotic curvature to orthotic intervention: when should a radiograph be obtained after

putting on or taking off a spinal orthosis? Spine (Phila Pa 1976). 2014;39(17):1408-16. doi: 10.1097/BRS.0000000000000423. PubMed PMID: 24859575.

24. Shi B, Guo J, Mao S, Wang Z, Yu FW, Lee KM, et al. Curve Progression in Adolescent Idiopathic Scoliosis With a Minimum of 2 Years' Follow-up After Completed Brace Weaning With Reference to the SRS Standardized Criteria. Spine Deform. 2016;4(3):200-5. doi: 10.1016/j.jspd.2015.12.002. PubMed PMID: 27927503.

25. Negrini S, Donzelli S, Aulisa AG, et al. 2016 SOSORT guidelines: orthopaedic and rehabilitation treatment of idiopathic scoliosis during growth. Scoliosis Spinal Disord. 2018;13:3. doi:10.1186/s13013-017-0145-8

26. Cheung KM, Senkoylu A, Alanay A, Genc Y, Lau S, Luk KD. Reliability and concurrent validity of the adapted Chinese version of Scoliosis Research Society-22 (SRS-22) questionnaire. Spine. 2007;32(10):1141-5. Epub 2007/05/02. doi: 10.1097/01.brs.0000261562.48888.e3. PubMed PMID: 17471100

Supplementary 2 Statistical Plan

TITLE: A RANDOMIZED CONTROLLED TRIAL COMPARING GRADUAL AND IMMEDIATE BRACE WEANING FOR CLINICAL MANAGEMENT OF ADOLESCENT IDIOPATHIC SCOLIOSIS PATIENTS

DATA PROCESSING AND ANALYSIS

1. SAMPLE SIZE CALCULATION

The sample size of this clinical trial will be determined based on an independent sample *t*-test to detect a difference between the two groups (immediate and gradual bracing weaning) for the changes in Cobb angle. According to a previous study [1], the average increase in Cobb angle at 1 year after brace weaning was 3.5 degrees. Curve progression is observed in 43.5% of patients. At 2 years, the curve progression rate was 46.5% with an average increase of major curve Cobb angle of 5.1 degrees. Based on this result, we consider a difference of 2 degrees between the changes in Cobb angle as clinically relevant, with an equivalence margin of 0.4. Assuming a standard deviation of 6 degrees for the Cobb angle change, a sample of 128 per group is required to detect the difference of the statistical test with 80% power at a 0.05 level of significance. Accounting for 20% overall attrition rate at 1 year follow-up, we will recruit 308 AIS patients (154 in each group) for this prospective study.

2. STUDY OUTCOMES

Primary outcome was the change in major curve Cobb angle from baseline to 6-month, 12-month, and 24-month follow-up. Curve progression was examined as >5º increase of major Cobb angle between follow-up time points and baseline. Secondary outcomes were the change in truncal balance in truncal shift and listing (C7-central sacral vertebral line deviation) from baseline to 6-month, 12-month and 24-month follow-up.

Secondary outcomes also included the refined Scoliosis Research Society 22-item (SRS-22r) questionnaire total score, the EuroQol-5-dimension (EQ-5D) utility score and EuroQol-visual analogue scale (EQ-VAS) at all post-weaning time points.

All study outcome measures were studied at baseline (the time of weaning), post-weaning 6, 12 and 24 months.

Body weight, standing and sitting body height, arm span, and HRQoL measures were measured, and curve type (i.e. thoracic, thoracolumbar/ lumbar) and coronal Cobb angle were assessed from whole spine standing posteroanterior and lateral radiographs taken simultaneously at each visit as per clinical routine. Skeletal maturity was routinely determined at the time of weaning using Risser staging, distal radius and ulna (DRU) classification and Sanders staging. In addition to Cobb angle and truncal balance, other radiological parameters including T1 tilt, shoulder height, sagittal vertical axis (SVA), thoracic kyphosis (T5-12) and lumbar lordosis (L1-S1) were measured by two assessors independently. An intra-class correlation coefficient (ICC) with 95% confidence interval will be used to estimate the inter-assessor reliability.

Statistical analysis

All statistical analyses will be carried out using SPSS Statistics v. 28.0 (Armonk, NY, IBM Corp) and sample size calculation will be performed using G*Power 3.1.9.7. For descriptive statistics, means and standard deviations or medians and interquartile ranges will be presented for continuous variables, frequencies and percentages will be presented for categorical/ discrete variables. No imputation will be performed for missing data.

1. Primary Analysis

The primary analysis will be evaluated by two independent sample *t*-test to determine the significance of the differences in change of Cobb angle and truncal balance

between the two groups. The analysis will primarily be carried out according to intention-to-treat (ITT) and subordinately based on per-protocol (PP) principle. The ITT analysis will include any randomized patients with non-missing efficacy measures. The PP analysis will include all randomize patients in ITT who fulfil the criteria of PP population.

The PP population is defined as a subset of the ITT population. Patients will be included in the PP population if they have:

- no late report violations of entry criteria,
- no incorrect treatment group assigned,
- no major protocol deviation(s) that is/are clinically significant to efficacy outcome,
- no treatment during follow-up after weaning, and
- with sufficient treatment compliance.

Sufficient treatment compliance is defined as gradual brace weaning patients having completed at least 8 hours/night of brace-wear for >80% of the 6-month gradual weaning period. Brace-wear compliance (hours/day) for the 6-month gradual weaning period will be recorded by thermal sensor.

Both ITT and PP results will be reported for primary analyse.

2. Secondary Analyses

The secondary efficacy analyses will be based on one-way analysis of covariance (ANCOVA) with post-hoc Bonferroni correction for intergroup comparisons. The ANCOVA model is used to analyze the effect of brace weaning protocols on the outcomes of changes of major curve Cobb angles and truncal balance as well as HRQoL measures, whilst adjusting for baseline covariates. Baseline covariates will be identified through testing the relationships of baseline parameters including socio-demographics, body size, maturity parameters, and other baseline radiographic measurements with the study outcomes using correlation tests.

The comparison between groups will be carried out using the adjusted mean

difference between the study groups at all post-weaning time points. The estimated difference between the two weaning protocols will be displayed along with the associated 95% confidence interval and p-value. The analysis will be carried out according to intention-to-treat (ITT) and PP principle.

3. Subgroup Analyses

In subgroup analysis, number of patients with curve progression since brace weaning (>5° increase of major Cobb angle) at post-weaning 24 months in the PP population will be reported with percentages. Multivariable logistic regressions were used to examine specifically whether brace weaning protocol (immediate/gradual), skeletal maturity status at weaning (Risser staging, DRU grades and Sanders stages) and any significant baseline parameters were associating factors for curve progression.

Pearson's Chi-square test was used to compare the occurrence of progression/non-progression/rebound (with >5° increase of major Cobb angle at follow-up but major Cobb angle at weaning was less than prebrace major Cobb angle) between weaning protocol.
