# Supplementary material for: Immediate vs Gradual Brace Weaning Protocols in Adolescent Idiopathic Scoliosis: A Randomized Clinical Trial
Source: JAMA Pediatr. 2024 Jun 3;178(7):657–68. doi: 10.1001/jamapediatrics.2024.1484 (PMC11148786; doi:10.1001/jamapediatrics.2024.1484)
Supplement: Supplement 2. — eTable 1. Baseline profile of immediate and gradual weaning group eTable 2. Correlation of baseline parameters with changes of major curve Cobb angles for ITT population eTable 3. Correlation of baseline parameters with changes of truncal balance and other radiological parameters and health-related quality of life measures for intention-to- treat (ITT) population eTable 4. Intergroup comparison of primary outcome without imputation in (a) primary analysis without adjustment and (b) secondary analyses with covariate adjustment eTable 5. Comparison of baseline demographics and patient characteristics at the time of brace weaning for all randomized patients with and without ITT post-weaning 24- month follow-up eTable 6. Cross-tabulation of skeletal maturity status at weaning and brace outcomes of curve progression at post-weaning 24-months between immediate and gradual weaning protocols in the Intention-to-treat (ITT) and per-protocol (PP) population eTable 7. Logistic regression for curve progression at post-weaning 24 months for the per-protocol (PP) population eTable 8. Intergroup comparison of (a) change of truncal balance without covariate adjustment and (b) other radiological parameters with/without covariate adjustment eTable 9. Comparison of health-related quality of life measures between groups without adjustment eFigure. Counts of non-progression, progression and rebound of curves at post- weaning 24 months for the PP population [file jamapediatr-e241484-s002.pdf]

Supplemental Online Content

Cheung PWH, Chan OKO, Wu H, et al. Immediate vs gradual brace weaning protocols in adolescent idiopathic scoliosis: a randomized clinical trial. *JAMA Pediatr*. Published online June 3, 2024. doi:10.1001/jamapediatrics.2024.1484

eTable 1. Baseline profile of immediate and gradual weaning group
eTable 2. Correlation of baseline parameters with changes of major curve Cobb angles for ITT population
eTable 3. Correlation of baseline parameters with changes of truncal balance and other radiological parameters and health-related quality of life measures for intention-to- treat (ITT) population
eTable 4. Intergroup comparison of primary outcome without imputation in (a) primary analysis without adjustment and (b) secondary analyses with covariate adjustment
eTable 5. Comparison of baseline demographics and patient characteristics at the time of brace weaning for all randomized patients with and without ITT post-weaning 24- month follow-up
eTable 6. Cross-tabulation of skeletal maturity status at weaning and brace outcomes of curve progression at post-weaning 24-months between immediate and gradual weaning protocols in the Intention-to-treat (ITT) and per-protocol (PP) population
eTable 7. Logistic regression for curve progression at post-weaning 24 months for the per-protocol (PP) population
eTable 8. Intergroup comparison of (a) change of truncal balance without covariate adjustment and (b) other radiological parameters with/without covariate adjustment
eTable 9. Comparison of health-related quality of life measures between groups without adjustment
eFigure. Counts of non-progression, progression and rebound of curves at post- weaning 24 months for the PP population

This supplemental material has been provided by the authors to give readers additional information about their work.

© 2024 Cheung PWH et al. *JAMA Pediatrics*.

eTable 1. Baseline profile of immediate and gradual weaning group

Characteristics	Immediate weaning		Gradual weaning		*P* value
	n	193	n	176	
Sex, n (column %)					
Female	193	156 (80.8)	176	148 (84.1)	.411
Male		37 (19.2)		28 (15.9)	
Curve type, n (column %)					
Thoracic	193	38 (19.7)	176	43 (24.4)	.272
Thoracolumbar/ lumbar		155 (80.3)		133 (75.6)	
Cobb angle at weaning, mean (SD), degrees					
Major curve	193	30.1 (7.8)	176	29.3 (8.4)	.298
Minor curve #	145	24.3 (7.6)	137	23.4 (8.0)	.289
Duration of bracing, mean (SD), months	193	26.8 (12.8)	176	25.0 (12.7)	.192
For girls					
Age at weaning, mean (SD), yrs	156	14.8 (1.0)	148	14.5 (0.9)	.028*
Standing height, mean (SD), cm	156	160.1 (5.5)	148	160.4 (6.3)	.728
Sitting height, mean (SD), cm	156	86.9 (2.8)	148	87.1 (3.1)	.586
Arm span, mean (SD), cm	156	159.6 (6.7)	148	159.3 (7.2)	.669
Weight, mean (SD), kg	156	47.7 (5.9)	148	47.3 (6.1)	.631
BMI, mean (SD), kg/m²	156	18.6 (2.0)	148	18.4 (2.1)	.462
Months post-menarche (remaining patients were pre-menarche), mean (SD), months	132	30.3 (7.6)	124	29.0 (6.4)	.134
Skeletal maturity, n (column%), counts					
DRU classification	156		148		
R8		2 (1.3)		0	.654
R9		33 (21.2)		36 (24.3)	
R10		95 (60.9)		87 (58.8)	
R11		26 (16.7)		25 (16.9)	
U7	156	20 (12.8)	148	24 (16.2)	.645
U8		93 (59.6)		82 (55.4)	
U9		43 (27.6)		42 (28.4)	
Sanders staging	156		148		
6		1 (0.6)		2 (1.4)	.749
7A		20 (12.8)		24 (16.2)	
7B		115 (73.7)		104 (70.3)	
8		20 (12.8)		18 (12.2)	
For boys					
Age at weaning, mean (SD), yrs	37	15.8 (1.4)	28	15.8 (1.0)	.850
Standing height, mean (SD), cm	37	171.1 (6.5)	28	173.0 (7.0)	.271
Sitting height, mean (SD), cm	37	91.6 (3.7)	28	92.5 (3.6)	.361
Arm span, mean (SD), cm	37	171.7 (6.2)	28	174.2 (9.1)	.201
Weight, mean (SD), kg	37	53.5 (7.9)	28	56.6 (12.1)	.226
BMI, mean (SD), kg/m²	37	18.3 (2.4)	28	18.8 (3.3)	.415

© 2024 Cheung PWH et al. *JAMA Pediatrics*.

Characteristics	n	Immediate weaning	n	Gradual weaning	*P* value
For boys					
Skeletal maturity, n (column%), counts					
DRU classification	37		28		
R9		6 (16.2)		4 (14.3)	.810
R10		23 (62.2)		20 (71.4)	
R11		8 (21.6)		4 (14.3)	
U7	37	3 (8.1)	28	5 (17.9)	.523
U8		22 (59.5)		15 (53.6)	
U9		12 (32.4)		8 (28.0)	
Sanders staging	37		28		
6		1 (2.7%)		1 (3.6%)	.225
7A		2 (5.4%)		6 (21.4%)	
7B		27 (73.0%)		18 (64.3%)	
8		7 (18.9%)		3 (10.7%)	

Abbreviations: n: number of patients analyzed, %: percentage, SD: standard deviation, kg: kilograms, cm: centimetres, BMI: body mass index, m: metre, DRU classification: distal radius and ulna classification, R: radius grade, U: ulna grade

^ Paired-sample *t*-tests were used for comparing continuous variables between groups, while Chi-square tests (or Fisher's exact tests when expected cell count was <5) and z-test were used for column proportion and cell count comparison for nominal or ordinal variables, with adjusted P values by Bonferroni method

* Statistical significance at $P < .05$

Number of patients analyzed for minor curves only when minor curves were present

© 2024 Cheung PWH et al. *JAMA Pediatrics*.

eTable 2. Correlation of baseline parameters with changes of major curve Cobb angles for ITT population

Baseline parameters	n	Change of major curve Cobb angles	
		Correlation coefficient [a]	*P* value
Age	369	-0.078	.156
Gender	369	0.011	.750
Curve type	369	0.071	.033*
Major Curve Cobb angles at weaning	369	-0.125	.044*
Risser stages	369	-0.042	.487
Radius grades	369	-0.073	.027*
Ulna grades	369	-0.210	< .001*
Sanders stages	369	-0.181	.003*
Bone age component score<br>(DRU grades and Sanders stage) [b]	369	-0.182	.003*

Abbreviation: n: number of patients analyzed, DRU: distal radius and ulna

[a] Spearman’s correlation test for continuous variable and point-biserial correlation test for dichotomous variable

[b] Avoid collinearity for the moderately to strongly correlated radius grades, ulna grades and Sanders stages using principal components analysis (PCA)

Sampling adequacy was acceptable (Kaiser-Meyer-Olkin Measure of Sampling Adequacy = .72) and Bartlett's Test of Sphericity demonstrated that correlations between items were large enough for PCA ($\chi^2(3) = 507.50$, $P < .001$).

* Statistical significance at $P < .05$

© 2024 Cheung PWH et al. *JAMA Pediatrics*.

eTable 3. Correlation of baseline parameters with changes of truncal balance and other radiological parameters and health-related quality of life measures for intention-to-treat (ITT) population

Baseline parameters	n	Change of truncal balance and other radiological parameters									
		Truncal shift		Listing		SVA		Thoracic kyphosis		Lumbar lordosis	
		r_s [a]	*P* value	r_s [a]	*P* value	r_s [a]	*P* value	r_s [a]	*P* value	r_s [a]	*P* value
Age	369	-0.050	.34	-0.039	.46	-0.048	.44	-0.022	.72	-0.029	.57
Gender	369	-0.062	.32	-0.044	.48	-0.054	.38	-0.047	.45	-0.032	.61
Curve type	369	0.063	.23	0.115	.027*	0.075	.23	-0.020	.74	-0.066	.29
Major Cobb angles at weaning	369	0.055	.38	0.073	.24	0.138	.026*	0.047	.45	0.098	.12
Risser stages	369	-0.021	.74	-0.022	.72	0.032	.61	-0.099	.11	0.011	.86
Radius grades	369	-0.069	.27	-0.016	.79	-0.132	.034*	-0.132	.03*	-0.089	.16
Ulna grades	369	-0.084	.19	-0.026	.66	-0.037	.51	-0.142	.01*	-0.077	.18
Sanders stages	369	-0.019	.76	0.037	.56	-0.111	.05	-0.074	.24	-0.129	.04*
Bone age component score	369	-0.070	.22	-0.020	.73	-0.083	.20	-0.168	.003*	-0.071	.27

Baseline parameters	n	Total SRS-22r scores		n	EQ-5D utility scores		n	EQ-VAS	
		Correlation coefficient [a]	*P* value		Correlation coefficient [a]	*P* value		Correlation coefficient [a]	*P* value
Age	350	-0.044	.46	352	0.049	.39	351	0.019	.74
Gender	350	-0.099	.06	352	0.023	.69	351	0.038	.51
Curve type	350	-0.108	.04*	352	-0.079	.13	351	-0.114	.04*
Major curve Cobb angles at weaning	350	-0.164	.002*	352	-0.095	.07	351	-0.219	<.001*
Risser stages	350	-0.040	.49	352	0.082	.12	351	0.065	.25
Radius grades	350	-0.091	.09	352	0.102	.05*	351	0.064	.23
Ulna grades	350	-0.129	.02*	352	0.072	.17	351	0.088	.10
Sanders stages	350	-0.016	.77	352	0.060	.30	351	0.046	.46
Bone age component score	350	0.131	.01*	352	0.023	.72	351	0.068	.29

Abbreviations: SVA: sagittal vertical axis, SRS-22r: refined Scoliosis Research Society 22-item, EQ-5D: EuroQol-5-dimension, EQ-VAS: EuroQol-visual analogue scale, DRU: distal radius and ulna

[a] Spearman's correlation test for continuous variable and point-biserial correlation test for dichotomous variable, r_s: Spearman's correlation coefficient

* Statistical significance at *P* < .05

© 2024 Cheung PWH et al. *JAMA Pediatrics*.

eTable 4. Intergroup comparison of primary outcome without imputation in (a) primary analysis without adjustment and (b) secondary analyses with covariate adjustment

Post-weaning time point	Immediate weaning (*I*)		Gradual weaning (*G*)		Mean difference *G - I* (95%CI)	*t / F*	η_p^2	*P* value
	n		n					
**(a) Primary analyses**								
ITT								
**Change of Cobb angle of major curve (vs baseline), mean (SD), degrees**								
6-months	182	2.0 (4.0)	169	1.4 (4.1)	-0.6 (-1.4, 0.3)	-1.307	N/A	.192
12-months	164	2.7 (4.7)	157	2.3 (4.4)	-0.4 (-1.4, 0.6)	-0.785	N/A	.433
24-months	147	3.5 (5.0)	137	2.9 (5.0)	-0.6 (-1.8, 0.5)	-1.082	N/A	.283
**Change of Cobb angle of minor curve (vs baseline) #, mean (SD) degrees**								
6-months	133	1.9 (4.4)	128	0.4 (4.4)	-1.5 (-2.6, -0.4)	-2.765	N/A	.006*
12-months	122	2.4 (4.8)	117	1.4 (4.5)	-1.0 (-2.2, 0.2)	-1.655	N/A	.099
24-months	108	1.9 (4.7)	99	0.9 (4.5)	-1.0 (-2.3, 0.3)	-1.568	N/A	.118
PP								
**Change of Cobb angle of major curve (vs baseline), mean (SD), degrees**								
6-months	175	2.1 (3.9)	163	1.4 (4.1)	-0.7 (-1.5, 0.2)	-1.582	N/A	.114
12-months	157	2.9 (4.5)	153	2.3 (4.4)	-0.6 (-1.6, 0.4)	-1.122	N/A	.263
24-months	141	3.5 (4.8)	133	2.9 (4.9)	-0.7 (-1.8, 0.5)	-1.129	N/A	.260
**Change of Cobb angle of minor curve (vs baseline) #, mean (SD) degrees**								
6-months	129	1.8 (4.4)	123	0.4 (4.2)	-1.4 (-2.5, -0.4)	-2.614	N/A	.009
12-months	118	2.4 (4.8)	114	1.4 (4.5)	-1.0 (-2.2, 0.2)	-1.564	N/A	.119
24-months	105	1.9 (4.6)	96	0.9 (4.5)	-1.0 (-2.3, 0.2)	-1.591	N/A	.113
**(b) Secondary analyses**								
ITT								
**Change of Cobb angle of major curve (vs baseline), adjusted mean (95% CI), SE, degrees**								
6-months	182	2.0<br>(1.4, 2.6), 0.3	169	1.4<br>(0.8, 2.0), 0.3	-0.6 (-1.4, 0.3)	1.816	0.005	.179
12-months	164	2.7<br>(2.0, 3.4), 0.4	157	2.3<br>(1.6, 3.1), 0.4	-0.4 (-1.4, 0.6)	0.619	0.002	.432
24-months	147	3.5<br>(2.7, 4.4), 0.4	137	2.9<br>(2.1, 3.7), 0.4	-0.7 (-1.8, 0.5)	1.193	0.004	.276
**Change of Cobb angle of minor curve (vs baseline) #, adjusted mean (95% CI), SE, degrees**								
6-months	133	1.8,<br>(1.1, 2.6), 0.4	128	0.4<br>(-0.4, 1.1), 0.4	-1.5 (-2.6, -0.4)	7.306	0.028	.007*
12-months	122	2.4<br>(1.6, 3.3), 0.4	117	1.5<br>(0.6, 2.3), 0.4	-1.0 (-2.2, 0.2)	2.640	0.011	.106
24-months	108	1.9<br>(1.1, 2.8), 0.4	99	0.9<br>(0.04, 1.85), 0.5	-1.0 (-2.2, 0.3)	2.466	0.011	.118
PP								
**Change of Cobb angle of major curve (vs baseline), adjusted mean (95% CI), SE, degrees**								
6-months	175	2.1<br>(1.5, 2.7), 0.3	163	1.4<br>(0.8, 2.0), 0.3	-0.7 (-1.6, 0.3)	2.780	0.008	.096
12-months	157	2.9<br>(2.2, 3.6), 0.4	153	2.3<br>(1.6, 3.0), 0.4	-0.6 (-1.6, 0.4)	1.346	0.004	.247
24-months	141	3.6<br>(2.8, 4.4), 0.4	133	2.9<br>(2.0, 3.7), 0.4	-0.7 (-1.9, 0.5)	1.355	0.005	.245
**Change of Cobb angle of minor curve (vs baseline) #, adjusted mean (95% CI), SE, degrees**								
6-months	129	1.8<br>(1.1, 2.6), 0.4	123	0.4<br>(-0.4, 1.2), 0.4	-1.4 (-2.5, -0.3)	6.478	0.026	.012
12-months	118	2.4<br>(1.5, 3.2), 0.4	114	1.4<br>(0.6, 2.3), 0.4	-1.0 (-2.2, 0.3)	2.402	0.010	.123
24-months	105	1.9<br>(1.0, 2.8), 0.4	96	0.9<br>(0.0, 1.8), 0.5	-1.0 (-2.3, 0.3)	2.473	0.012	.117

© 2024 Cheung PWH et al. *JAMA Pediatrics*.

Abbreviations: n: number of patients analyzed, SE: standard error, CI: confidence interval, N/A: not applicable for primary analyses
η_p^2: Partial eta squared
[a] mean difference for primary analyses, and mean difference based on adjusted estimated marginal means for secondary analyses
t: t-statistic value for independent samples t-test with Levene's test for primary analyses
F: F ratio adjusted for covariates (Major curve Cobb angles, curve types and bone age at weaning) in ANCOVA analyses for secondary analyses
* Statistical significance at Bonferroni-adjusted $P < .008$
# Number of patients analyzed for minor curves only when minor curves were present

© 2024 Cheung PWH et al. *JAMA Pediatrics*.

eTable 5. Comparison of baseline demographics and patient characteristics at the time of brace weaning for all randomized patients with and without ITT post-weaning 24-month follow-up

Characteristics	ITT with post-weaning 24-month follow-up		ITT without post-weaning 24-month follow-up		*P* value
	n	284	n	85	
Sex, n (column %)					
Female	284	239 (84.2)	85	65 (76.5)	.103
Male		45 (15.8)		20 (23.5)	
Curve type, n (column %)					
Thoracic	284	63 (22.2)	85	18 (21.2)	.844
Thoracolumbar/ lumbar		221 (77.8)		67 (78.8)	
Cobb angle at weaning, mean (SD), degrees					
Major curve	284	30.1 (8.3)	85	28.4 (7.6)	.088
Minor curve #	223	24.3 (7.7)	59	22.2 (7.8)	.067
Duration of bracing, mean (SD), months	284	26.5 (13.5)	85	24.0 (10.0)	.124
For girls					
Age at weaning, mean (SD), yrs	239	14.7 (1.0)	65	14.6 (1.1)	.767
Standing height, mean (SD), cm	239	160.4 (6.0)	65	159.8 (5.4)	.515
Sitting height, mean (SD), cm	239	87.0 (2.9)	65	87.0 (3.1)	.929
Arm span, mean (SD), cm	239	159.6 (7.1)	65	159.0 (6.3)	.544
Weight, mean (SD), kg	239	47.6 (6.0)	65	47.3 (6.1)	.685
BMI, mean (SD), kg/m²	239	18.5 (1.9)	65	18.5 (2.3)	.988
Months post-menarche (remaining patients were pre-menarche), mean (SD), months	203	29.5 (6.9)	53	30.2 (7.6)	.546
Skeletal maturity, n (column%), counts					
DRU classification<br>R8<br>R9<br>R10<br>R11	239	 2 (0.8)<br>55 (23.0)<br>144 (60.3)<br>38 (15.9)	65	 0<br>14 (21.5)<br>38 (58.5)<br>13 (20.0)	.829
U7<br>U8<br>U9	239	38 (15.9)<br>135 (56.5)<br>66 (27.6)	65	6 (9.2)<br>40 (61.5)<br>19 (29.2)	.398
Sanders staging<br>6<br>7A<br>7B<br>8	239	 3 (1.3)<br>38 (15.9)<br>169 (70.7)<br>29 (12.1)	65	 0<br>6 (9.2)<br>50 (76.9)<br>9 (13.8)	.515
For boys					
Age at weaning, mean (SD), yrs	45	15.8 (1.3)	20	15.7 (1.1)	.790
Standing height, mean (SD), cm	45	171.9 (6.3)	20	172.1(7.8)	.903
Sitting height, mean (SD), cm	45	91.9 (3.4)	20	92.1 (4.3)	.838
Arm span, mean (SD), cm	45	173.3 (7.6)	20	171.7 (7.8)	.422
Weight, mean (SD), kg	45	54.7 (11.0)	20	55.2 (7.3)	.858
BMI, mean (SD), kg/m²	45	18.5 (3.1)	20	18.6 (2.3)	.812
Skeletal maturity, n (column%), counts					
DRU classification<br>R9<br>R10<br>R11	45	 8 (17.8)<br>31 (68.9)<br>6 (13.3)	20	 2 (10.0)<br>12 (60.0)<br>6 (30.0)	.298
U7<br>U8<br>U9	45	6 (13.3)<br>28 (62.2)<br>11 (24.4)	20	2 (10.0)<br>9 (45.0)<br>9 (45.0)	.247

© 2024 Cheung PWH et al. *JAMA Pediatrics*.

Characteristics	n	ITT with post-weaning 24-month follow-up	n	ITT without post-weaning 24-month follow-up	*P* value
For boys					
Sanders staging	45		20		.190
6		2 (4.4)		0	
7A		6 (13.3)		2 (10.0)	
7B		33 (73.3)		12 (60.0)	
8		4 (8.9)		6 (30.0)	

Abbreviations: n: number of patients analyzed, %: percentage, SD: standard deviation, kg: kilograms, cm: centimetres, BMI: body mass index, m: metre, DRU classification: distal radius and ulna classification, R: radius grade, U: ulna grade

^ Paired-sample *t*-tests were used for comparing continuous variables between groups, while Chi-square tests (or Fisher's exact tests when expected cell count was <5) and z-test were used for column proportion and cell count comparison for nominal or ordinal variables, with adjusted P values by Bonferroni method

* Statistical significance at $P < .05$

Number of patients analyzed for minor curves only when minor curves were present

© 2024 Cheung PWH et al. *JAMA Pediatrics*.

eTable 6. Cross-tabulation of skeletal maturity status at weaning and brace outcomes of curve progression at post-weaning 24-months between immediate and gradual weaning protocols in the Intention-to-treat (ITT) and per-protocol (PP) population

	ITT (n=369)				PP (n=365)			
	Immediate weaning n = 193		Gradual weaning n=176		Immediate weaning n = 191		Gradual weaning n=174	
Lost to 24-month FU	46		39		46		37	
Attended	147		137		145		137	
	Progress -ion (n = 25)	Non-progress -ion (n = 122)	Progress -ion (n = 29)	Non-progress -ion (n = 108)	Progress -ion (n = 24)	Non-progress -ion (n = 121)	Progress -ion (n = 29)	Non-progress -ion (n = 108)
Skeletal maturity grading at weaning, in row % per grade (n)								
Risser staging								
4	14.6% (12/82)	85.4% (70/82)	26.8% (19/71)	73.2% (52/71)	13.75% (11/80)	86.25% (69/80)	26.8% (19/71)	73.2% (52/71)
4+	33.3% (9/27)	66.7% (18/27)	9.8% (4/41)	90.2% (37/41)	33.3% (9/27)	66.7% (18/27)	9.8% (4/41)	90.2% (37/41)
5	10.5% (4/38)	89.5% (34/38)	24.0% (6/25)	76.0% (19/25)	10.5% (4/38)	89.5% (34/38)	24.0% (6/25)	76.0% (19/25)
DRU Classification Radius grades								
R8	0% (0/2)	100% (2/2)	n=0	n=0	0% (0/2)	100% (2/2)	n=0	n=0
R9	25.0% (7/28)	75.0% (21/28)	31.4% (11/35)	68.6% (24/35)	22.2 (6/27)	77.8% (21/27)	31.4% (11/35)	68.6% (24/35)
R10	17.6% (16/91)	82.4% (75/91)	20.2% (17/84)	79.8% (67/84)	17.8% (16/90)	82.2% (74/90)	20.2% (17/84)	79.8% (67/84)
R11	7.7% (2/26)	92.3% (24/26)	5.6% (1/18)	94.4% (17/18)	7.7% (2/26)	92.3% (24/26)	5.6% (1/18)	94.4% (17/18)
Ulna grades								
U7	36.8% (7/19)	63.2% (12/19)	29.2% (7/24)	70.8% (17/24)	33.3% (6/18)	66.7% (12/18)	29.2% (7/24)	70.8% (17/24)
U8	17.2% (15/87)	82.8% (72/87)	24.7% (19/77)	75.3% (58/77)	17.4% (15/86)	82.6% (71/86)	24.7% (19/77)	75.3% (58/77)
U9	7.3% (3/41)	92.7% (38/41)	8.3% (3/36)	91.7% (33/36)	7.3% (3/41)	92.7% (38/41)	8.3% (3/36)	91.7% (33/36)
Sanders staging								
SS6	50.0% (1/2)	50.0% (1/2)	66.7% (2/3)	33.3% (1/3)	0% (0/1)	100% (1/1)	66.7% (2/3)	33.3% (1/3)
SS7a	38.9% (7/18)	61.1% (11/18)	24.0% (6/25)	76.0% (19/25)	38.9% (7/18)	61.1% (11/18)	24.0% (6/25)	76.0% (19/25)
SS7b	15.0% (16/107)	85.0% (91/107)	20.8% (20/96)	79.2% (76/96)	15.1% (16/106)	84.9% (90/106)	20.8% (20/96)	79.2% (76/96)
SS8	5.0% (1/20)	95.0% (19/20)	7.7% (1/13)	92.3% (12/13)	5.0% (1/20)	95.0% (19/20)	7.7% (1/13)	92.3% (12/13)

Abbreviations: %: percentage, n: number, DRU: distal radius and ulna, FU: follow-up

© 2024 Cheung PWH et al. *JAMA Pediatrics*.

eTable 7. Logistic regression for curve progression at post-weaning 24 months for the per-protocol (PP) population

Radius grades at weaning					
	χ^2 (5) = 34.444, *P*< .001 Post-weaning 24-month follow-up n=282 of PP cohort (n=365)				
Parameters	**B**	**S.E.**	**Wald**	***P* value**	**OR (95% CI)**
Protocol (Ref: gradual weaning)	-0.332	0.329	1.020	.31	0.72 (0.38, 1.37)
Radius grades at weaning (Ref: R11)			9.854	0.007*	
R8[a]/R9	2.097	0.700	8.980	.003*	8.14 (2.07, 32.07)
R10	1.339	0.651	4.230	.04*	3.81 (1.07, 13.66)
Weaning major Cobb angles	0.102	0.022	21.770	< .001*	1.11 (1.06, 1.16)
Curve type (Ref: TL/L)	-0.187	0.411	0.207	.65	0.83 (0.37, 1.86)
[a] R8: n=2					
Ulna grades at weaning					
	χ^2 (5) = 39.329, *P*< .001 Post-weaning 24-month follow-up n=282 of PP cohort (n=365)				
Parameters	**B**	**S.E.**	**Wald**	***P* value**	**OR (95% CI)**
Protocol (Ref: gradual weaning)	-0.393	0.333	1.396	.24	0.68 (0.35, 1.30)
Ulna grades at weaning (Ref: U9)			13.292	.001*	
U7	2.109	0.589	12.836	< .001*	8.24 (2.60, 26.14)
U8	1.520	0.498	9.293	.002*	4.57 (1.72, 12.14)
Weaning major Cobb angles	0.103	0.022	21.989	< .001*	1.11 (1.06, 1.16)
Curve type (Ref: TL/L)	-0.297	0.419	0.505	.48	0.74 (0.33, 1.69)
Sanders stages at weaning					
	χ^2 (6) = 36.606, *P*< .001 Post-weaning 24-month follow-up n=282 of PP cohort (n=365)				
Parameters	**B**	**S.E.**	**Wald**	***P* value**	**OR (95% CI)**
Protocol (Ref: gradual weaning)	-0.304	0.331	0.843	.36	0.74 (0.39, 1.41)
Sanders stages at weaning (Ref: SS8)			12.419	.006*	
SS6	3.873	1.338	8.377	.004*	48.09 (3.49, 662.44)
SS7a	2.270	0.841	7.282	.007*	9.68 (1.86, 50.35)
SS7b	1.451	0.777	3.486	.06	4.27 (0.93, 19.57)
Weaning major Cobb angles	0.102	0.022	21.753	< .001*	1.11 (1.06, 1.16)
Curve type (Ref: TL/L)	-0.155	0.418	0.137	.71	0.86 (0.38, 1.94)
Risser stages at weaning					
	χ^2 (5) = 24.128, *P*< .001 Post-weaning 24-month follow-up n=282 of PP cohort (n=365)				
Parameters	**B**	**S.E.**	**Wald**	***P* value**	**OR (95% CI)**
Protocol (Ref: gradual weaning)	-0.422	0.324	1.698	.19	0.68 (0.35, 1.24)
Risser stages at weaning (Ref: 5)			1.498	.47	
4	0.471	0.425	1.229	.27	1.60 (0.70, 3.69)
4+	0.149	0.488	0.093	.76	1.16 (0.45, 3.02)
Weaning major Cobb angles	0.092	0.021	19.770	< .001*	1.10 (1.05, 1.14)
Curve type (Ref: TL/L)	-0.09	0.405	0.053	.82	0.91 (0.41, 2.01)

Abbreviations: n: number of patients analyzed, B: unstandardized coefficient, S.E. Standard error, Wald: wald test
Ref: reference group, TL/L: Thoracolumbar/lumbar
* Statistical significance at *P* < .05

© 2024 Cheung PWH et al. *JAMA Pediatrics*.

eTable 8. Intergroup comparison of (a) change of truncal balance without covariate adjustment and (b) other radiological parameters with/without covariate adjustment

Post-weaning time point	Immediate weaning (*I*)		Gradual weaning (*G*)		Mean difference[a] *G - I* (95%CI)	*t / F*	η_p^2	*P* value ^
**(a) Truncal balance (without adjustment)^**								
ITT								
**Change of truncal shift (vs baseline), mean (SD), mm**								
	n		n					
6-months	193	0.9 (7.6)	176	1.1 (8.4)	0.2 (-1.4, 1.8)	0.240	N/A	.810
12-months	193	1.5 (8.1)	176	2.3 (8.9)	0.8 (-0.9, 2.6)	0.938	N/A	.349
24-months	193	2.5 (9.2)	176	2.3 (9.3)	-0.2 (-2.1, 1.7)	-0.211	N/A	.833
**Change of listing (vs baseline), mean (SD), mm**								
6-months	193	0.5 (9.0)	176	0.7 (9.9)	0.1 (-1.8, 2.1)	0.142	N/A	.877
12-months	193	0.2 (9.6)	176	1.0 (10.2)	0.8 (-1.2, 2.8)	0.756	N/A	.450
24-months	193	2.2 (10.0)	176	2.1 (11.7)	-0.1 (-2.4, 2.1)	-0.100	N/A	.921
PP								
**Change of truncal shift (vs baseline), mean (SD), mm**								
6-months	186	0.8 (7.6)	169	1.2 (8.1)	0.4 (-1.2, 2.1)	0.526	N/A	.599
12-months	186	1.3 (7.7)	169	2.5 (8.8)	1.2 (-0.6, 2.9)	1.336	N/A	.182
24-months	186	2.4 (9.2)	169	2.5 (9.0)	0.2 (-1.7, 2.0)	0.157	N/A	.875
**Change of listing (vs baseline), mean (SD), mm**								
6-months	186	0.5 (9.0)	169	0.7 (9.8)	0.2 (-1.8, 2.2)	0.188	N/A	.851
12-months	186	-0.05 (9.44)	169	1.2 (10.1)	1.2 (-0.8, 3.3)	1.171	N/A	.242
24-months	186	2.1 (10.0)	169	2.2 (11.4)	0.2 (-2.1, 2.4)	0.146	N/A	.884
**(b) Other radiological parameters (with adjustment)** ⟊								
ITT								
**Change of T1-tilt (vs baseline), adjusted mean (SE), degrees**								
6-months	193	-0.6 (0.2)	176	-0.1 (0.2)	0.5 (-0.1, 1.1)	3.044	0.008	.082
12-months	193	-0.7 (0.2)	176	-0.4 (0.2)	0.4 (-0.3, 1.0)	1.215	0.003	.271
24-months	193	-0.4 (0.2)	176	-0.2 (0.3)	0.2 (-0.5, 0.9)	0.239	0.001	.625
**Change of shoulder height (vs baseline), adjusted mean (SE), mm**								
6-months	193	0.04 (0.69)	176	0.84 (0.72)	0.80 (-1.16, 2.76)	0.642	0.002	.424
12-months	193	0.6 (0.8)	176	0.8 (0.8)	0.2 (-2.0, 2.4)	0.029	0.000	.866
24-months	193	-0.2 (0.7)	176	0.3 (0.8)	0.5 (-1.6, 2.6)	0.215	0.001	.643
**Change of SVA (vs baseline), adjusted mean (SE), mm**								
6-months	193	-0.9 (1.5)	176	3.0 (1.6)	4.0 (-0.4, 8.3)	3.258	0.009	.072
12-months	193	0.8 (1.5)	176	4.3 (1.6)	3.4 (-0.9, 7.8)	2.409	0.007	.121
24-months	193	-1.1 (1.5)	176	2.8 (1.5)	3.9 (-0.2, 8.1)	3.480	0.009	.063
**Change of thoracic kyphosis (vs baseline), adjusted mean (SE), degrees**								
6-months	193	-0.1 (0.6)	176	0.2 (0.6)	0.3 (-1.3, 1.9)	0.147	0.000	.702
12-months	193	0.3 (0.6)	176	0.7 (0.6)	0.4 (-1.3, 2.1)	0.236	0.001	.627
24-months	193	0.6 (0.7)	176	1.8 (0.7)	1.2 (-0.6, 3.1)	1.677	0.005	.196
**Change of lumbar lordosis (vs baseline), adjusted mean (SE), degrees**								
6-months	193	-0.4 (0.6)	176	-0.01 (0.59)	0.4 (-1.2, 2.0)	0.215	0.001	.643
12-months	193	-0.3 (0.6)	176	0.2 (0.7)	0.5 (-1.3, 2.3)	0.303	0.001	.582
24-months	193	1.1 (0.7)	176	0.9 (0.8)	-0.2 (-2.4, 1.9)	0.050	0.000	.823
PP								
**Change of T1-tilt (vs baseline), adjusted mean (SE), degrees**								
6-months	186	-0.6 (0.2)	169	-0.1(0.2)	0.5 (-0.1, 1.1)	2.350	0.007	.126
12-months	186	-0.7 (0.2)	169	-0.4 (0.2)	0.4 (-0.3, 1.0)	1.175	0.003	.279
24-months	186	-0.4 (0.2)	169	-0.2 (0.3)	0.2 (-0.5, 0.9)	0.289	0.001	.591
**Change of shoulder height (vs baseline), adjusted mean (SE), mm**								
6-months	186	0.1 (0.7)	169	0.8 (0.7)	0.7 (-1.3, 2.7)	0.509	0.001	.476
12-months	186	0.6 (0.8)	169	0.8 (0.8)	0.1 (-2.1, 2.4)	0.015	0.000	.901
24-months	186	-0.4 (0.8)	169	0.3 (0.8)	0.7 (-1.4, 2.9)	0.440	0.001	.508

© 2024 Cheung PWH et al. *JAMA Pediatrics*.

eTable 8. Intergroup comparison of (b) other radiological parameters with adjustment (cont'd)								
Post-weaning time point	n	Immediate weaning (*I*)	n	Gradual weaning (*G*)	Mean difference [a] *G - I* (95%CI)	*t / F*	η_p^2	*P* value ^
**Change of SVA (vs baseline), adjusted mean (SE), mm**								
6-months	186	-0.7 (1.5)	169	2.8 (1.6)	3.4 (-1.0, 7.8)	2.371	0.007	.124
12-months	186	1.3 (1.5)	169	4.1 (1.6)	2.8 (-1.5, 7.2)	1.623	0.005	.203
24-months	186	-0.9 (1.5)	169	2.8 (1.5)	3.7 (-0.5, 8.0)	2.981	0.008	.085
**Change of thoracic kyphosis (vs baseline), adjusted mean (SE), degrees**								
6-months	186	-0.3 (0.6)	169	0.1 (0.6)	0.4 (-1.2, 2.0)	0.261	0.001	.610
12-months	186	0.2 (0.6)	169	0.5 (0.6)	0.3 (-1.4, 2.0)	0.112	0.000	.738
24-months	186	0.3 (0.7)	169	1.7 (0.7)	1.4 (-0.6, 3.3)	0.749	0.006	.162
**Change of lumbar lordosis (vs baseline), adjusted mean (SE), degrees**								
6-months	186	0.01 (0.57)	169	-0.05 (0.59)	-0.06 (-1.68, 1.55)	0.006	0.000	.939
12-months	186	0.07 (0.63)	169	0.15 (0.66)	0.08 (-1.71, 1.87)	0.007	0.000	.932
24-months	186	1.6 (0.8)	169	1.0 (0.8)	-0.6 (-2.8, 1.6)	0.303	0.001	.582
**Other radiological parameters without adjustment^**								
ITT								
**Change of T1-tilt (vs baseline), mean (SD), degrees**								
6-months	193	-0.5 (3.1)	176	-0.1 (2.6)	0.5 (-0.1, 1.1)	1.512	N/A	.131
12-months	193	-0.7 (3.3)	176	-0.4 (2.8)	0.3 (-0.3, 0.9)	0.962	N/A	.337
24-months	193	-0.4 (3.4)	176	-0.3 (3.3)	0.1 (-0.6, 0.8)	0.274	N/A	.785
**Change of shoulder height (vs baseline), mean (SD), mm**								
6-months	193	0.1 (9.8)	176	0.8 (9.2)	0.8 (-1.2, 2.7)	0.779	N/A	.437
12-months	193	0.6 (10.6)	176	0.8 (10.9)	0.2 (-2.0, 2.4)	0.157	N/A	.876
24-months	193	-0.3 (9.6)	176	0.4 (11.2)	0.7 (-1.5, 2.8)	0.619	N/A	.536
**Change of SVA (vs baseline), mean (SD), mm**								
6-months	193	-0.9 (20.1)	176	2.9 (21.7)	3.8 (-0.5, 8.0)	1.726	N/A	.085
12-months	193	0.9 (20.6)	176	4.3 (21.7)	3.4 (-0.9, 7.7)	1.550	N/A	.122
24-months	193	-1.1 (19.7)	176	2.8 (20.5)	3.9 (-0.2, 8.0)	1.866	N/A	.063
**Change of thoracic kyphosis (vs baseline), mean (SD), degrees**								
6-months	193	-0.1 (6.4)	176	0.2 (8.9)	0.3 (-1.3, 1.9)	0.388	N/A	.699
12-months	193	0.2 (6.8)	176	0.7 (9.7)	0.4 (-1.3, 2.2)	0.518	N/A	.605
24-months	193	0.6 (8.0)	176	1.7 (10.1)	1.1 (-0.7, 3.0)	1.212	N/A	.226
**Change of lumbar lordosis (vs baseline), mean (SD), degrees**								
6-months	193	-0.4 (7.5)	176	0.02 (8.15)	0.4 (-1.2, 2.0)	0.439	N/A	.661
12-months	193	-0.3 (8.8)	176	0.2 (8.5)	0.5 (-1.3, 2.3)	0.563	N/A	.574
24-months	193	1.1 (10.7)	176	0.9 (9.7)	-0.2 (-2.2, 1.9)	-0.150	N/A	.881
PP								
**Change of T1-tilt (vs baseline), mean (SD), degrees**								
6-months	186	-0.5 (3.2)	169	-0.1 (2.6)	0.4 (-0.2, 1.0)	1.280	N/A	.201
12-months	186	-0.7 (3.3)	169	-0.4 (2.8)	0.3 (-0.3, 1.0)	0.934	N/A	.351
24-months	186	-0.4 (3.4)	169	-0.3 (3.3)	0.1 (-0.6, 0.8)	0.302	N/A	.763
**Change of shoulder height (vs baseline), mean (SD), mm**								
6-months	186	0.1 (9.8)	169	0.8 (9.3)	0.7 (-1.3, 2.7)	0.677	N/A	.499
12-months	186	0.6 (10.5)	169	0.8 (11.0)	0.1 (-2.1, 2.4)	0.125	N/A	.901
24-months	186	-0.5 (9.4)	169	0.4 (11.3)	0.9 (-1.2, 3.1)	0.844	N/A	.400
**Change of SVA (vs baseline), mean (SD), mm**								
6-months	186	-0.6 (19.8)	169	2.7 (21.9)	3.2 (-1.1, 7.6)	1.456	N/A	.146
12-months	186	1.3 (20.1)	169	4.1 (21.6)	2.9 (-1.5, 7.2)	1.288	N/A	.199
24-months	186	0.9 (19.6)	169	2.8 (20.6)	3.7 (-0.5, 7.9)	1.747	N/A	.081

© 2024 Cheung PWH et al. *JAMA Pediatrics*.

eTable 8. Intergroup comparison of (b) other radiological parameters with adjustment (cont'd)								
Post-weaning time point	n	Immediate weaning (*I*)	n	Gradual weaning (*G*)	Mean difference [a] *G - I* (95%CI)	*t* / *F*	η_p^2	*P* value ^
**Change of thoracic kyphosis (vs baseline), mean (SD), degrees**								
6-months	186	-0.3 (6.3)	169	0.1 (8.9)	0.4 (-1.2, 2.0)	0.550	N/A	.583
12-months	186	0.2 (6.9)	169	0.5 (9.7)	0.4 (-1.4, 2.1)	0.409	N/A	.683
24-months	186	0.4 (7.9)	169	1.6 (10.2)	1.3 (-0.6, 3.2)	1.332	N/A	.184
**Change of lumbar lordosis (vs baseline), mean (SD), degrees**								
6-months	186	0.02 (7.25)	169	-0.06 (8.11)	-0.1 (-1.7, 1.5)	-0.100	N/A	.920
12-months	186	0.1 (8.5)	169	0.2 (8.5)	0.1 (-1.7, 1.9)	0.089	N/A	.929
24-months	186	1.5 (10.6)	169	1.0 (9.8)	-0.5 (-2.7, 1.6)	-0.485	N/A	.628

Abbreviations: SD: standard deviation, mm: millimetres, SVA: sagittal vertical axis, SE: standard error, CI: confidence interval, N/A: not applicable for primary analyses

η_p^2: Partial eta squared

t: t-statistic value for independent samples t-test with Levene's test for primary analyses without covariate adjustment

F: F ratio adjusted for covariates (Major curve Cobb angles, curve types and bone age gradings at weaning) in ANCOVA for secondary analyses

[a] mean difference for primary analyses; or mean difference based on adjusted estimated marginal means for secondary analyses with covariate adjustment

^ Statistical significance at Bonferroni-adjusted *P* < .008 for truncal balance parameters

‡ Statistical significance at Bonferroni-adjusted *P* < .002 for other radiological parameters

© 2024 Cheung PWH et al. *JAMA Pediatrics*.

eTable 9. Comparison of health-related quality of life measures between groups without adjustment

Post-weaning time points	n	Immediate weaning (*I*)	n	Gradual weaning (*G*)	*Z* [a]	*P* value
ITT						
**SRS-22r domain scores, median (IQR)**						
Function						
6-months	193	5.00 (4.80, 5.00)	176	5.00 (4.80, 5.00)	-1.200	.230
12-months	193	5.00 (4.80, 5.00)	176	5.00 (4.85, 5.00)	-0.113	.910
24-months	193	5.00 (4.90, 5.00)	176	5.00 (4.80, 5.00)	-0.687	.492
Pain						
6-months	193	4.90 (4.60, 5.00)	176	4.80 (4.60, 5.00)	-0.640	.522
12-months	193	5.00 (4.60, 5.00)	176	5.00 (4.60, 5.00)	-0.032	.974
24-months	193	4.80 (4.50, 5.00)	176	4.80 (4.50, 5.00)	-0.439	.661
Self-image						
6-months	193	4.00 (3.60, 4.50)	176	3.80 (3.40, 4.40)	-1.660	.097
12-months	193	4.20 (3.60, 4.60)	176	4.10 (3.70, 4.50)	-0.208	.835
24-months	193	4.00 (3.60, 4.35)	176	4.10 (3.75, 4.45)	1.880	.060
Mental health						
6-months	193	4.40 (4.00, 5.00)	176	4.40 (4.00, 5.00)	0.225	.822
12-months	193	4.40 (4.00, 4.80)	176	4.60 (4.00, 5.00)	1.090	.276
24-months	193	4.20 (4.00, 4.70)	176	4.40 (4.00, 4.80)	1.108	.268
Satisfaction with treatment#						
6-months	76	4.00 (3.50, 4.50)	110	4.00 (4.00, 4.50)	0.766	.444
12-months	63	4.00 (3.50, 4.50)	81	4.00 (4.00, 4.50)	-0.066	.947
24-months	74	4.00 (3.50, 4.50)	71	4.00 (4.00, 4.50)	0.500	.617
**SRS-22r total scores, median (IQR)**						
6-months	193	4.50 (4.30, 4.74)	176	4.45 (4.24, 4.68)	-1.290	.197
12-months	193	4.55 (4.34, 4.75)	176	4.50 (4.30, 4.75)	0.052	.959
24-months	193	4.45 (4.31, 4.60)	176	4.50 (4.35, 4.65)	1.116	.265
**EQ-5D Utility scores, median (IQR)**						
6-months	193	1.000 (0.933, 1.000)	176	1.000 (0.950, 1.000)	-0.049	.961
12-months	193	1.000 (0.930, 1.000)	176	1.000 (0.948, 1.000)	-0.091	.927
24-months	193	1.000 (1.000, 1.000)	176	1.000 (1.000, 1.000)	0.102	.919
**EQ-VAS, median (IQR)**						
6-months	193	88.0 (80.0, 95.0)	176	90.0 (80.0, 95.0)	-0.122	.903
12-months	193	90.0 (80.0, 95.0)	176	90.0 (80.0, 95.0)	0.670	.503
24-months	193	87.0 (80.0, 93.0)	176	86.0 (80.0, 95.0)	0.527	.598
PP						
**SRS-22r domain scores, median (IQR)**						
Function						
6-months	186	5.00 (4.80, 5.00)	169	5.00 (4.80, 5.00)	-1.362	.173
12-months	186	5.00 (4.85, 5.00)	169	5.00 (4.90, 5.00)	0.106	.916
24-months	186	5.00 (4.90, 5.00)	169	5.00 (4.80, 5.00)	-0.981	.327
Pain						
6-months	186	5.00 (4.60, 5.00)	169	4.80 (4.60, 5.00)	-0.553	.580
12-months	186	5.00 (4.60, 5.00)	169	5.00 (4.60, 5.00)	0.006	.995
24-months	186	4.80 (4.50, 5.00)	169	4.80 (4.60, 5.00)	-0.206	.836
Self-image						
6-months	186	4.10 (3.60, 4.45)	169	3.80 (3.40, 4.40)	-1.760	.078
12-months	186	4.20 (3.70, 4.55)	169	4.00 (3.60, 4.45)	-0.390	.697
24-months	186	4.00 (3.60, 4.35)	169	4.10 (3.75, 4.50)	1.650	.099
Mental health						
6-months	186	4.40 (4.00, 5.00)	169	4.40 (4.00, 5.00)	0.068	.946
12-months	186	4.40 (4.00, 4.80)	169	4.60 (4.00, 5.00)	1.016	.310
24-months	186	4.30 (4.00, 4.70)	169	4.40 (4.00, 4.80)	1.004	.315
eTable 9. Comparison of health-related quality of life measures between groups without adjustment (continued)						
Post-weaning time points	n	Immediate weaning (*I*)	n	Gradual weaning (*G*)	*Z* [a]	*P* value

© 2024 Cheung PWH et al. *JAMA Pediatrics*.

Satisfaction with treatment#, median (IQR)						
6-months	72	4.00 (3.50, 4.50)	108	4.00 (4.00, 4.50)	0.575	.565
12-months	59	4.00 (4.00, 4.50)	79	4.00 (4.00, 4.50)	-0.322	.748
24-months	71	4.00 (3.50, 4.50)	70	4.00 (4.00, 4.50)	0.487	.626
**SRS-22r total scores, median (IQR)**						
6-months	186	4.50 (4.32, 4.74)	169	4.45 (4.24, 4.68)	-1.386	.166
12-months	186	4.55 (4.35, 4.75)	169	4.50 (4.30, 4.75)	0.071	.943
24-months	186	4.50 (4.33, 4.60)	169	4.50 (4.36, 4.65)	1.159	.247
**EQ-5D Utility scores, median (IQR)**						
6-months	186	1.000 (0.983, 1.000)	169	1.000 (1.000, 1.000)	-0.023	.981
12-months	186	1.000 (0.930, 1.000)	169	1.000 (1.000, 1.000)	0.061	.951
24-months	186	1.000 (1.000, 1.000)	169	1.000 (1.000, 1.000)	0.086	.931
**EQ-VAS, median (IQR)**						
6-months	186	88.0 (80.0, 95.0)	169	90.0 (80.0, 95.0)	0.193	.847
12-months	186	90.0 (80.0, 95.0)	169	90.0 (80.0, 96.0)	0.786	.432
24-months	186	87.0 (80.0, 94.0)	169	88.0 (80.0, 95.0)	0.632	.527

Abbreviations: n: number of patients analyzed, SRS-22r: refined Scoliosis Research Society 22-item, EQ-5D: EuroQol-5-dimension, EQ-VAS: EuroQol-visual analogue scale

[a] Mann-Whitney *U* test

* Statistical significance at $P < .002$

Patients could opt for answering questions for this domain if without treatment but not affecting calculation of the SRS total score, hence not subjected to multiple imputation

© 2024 Cheung PWH et al. *JAMA Pediatrics*.

eFigure. Counts of non-progression, progression and rebound of curves at post-weaning 24 months for the PP population

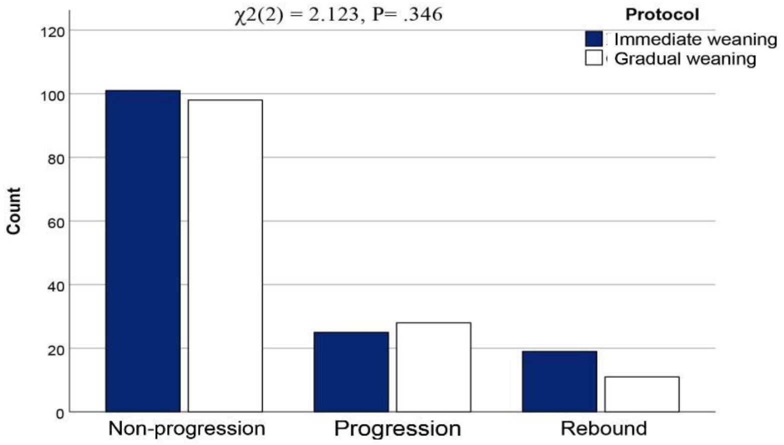

© 2024 Cheung PWH et al. *JAMA Pediatrics*.
