# Supplementary material for: Immediate vs Gradual Brace Weaning Protocols in Adolescent Idiopathic Scoliosis: A Randomized Clinical Trial
Source: JAMA Pediatr. 2024 Jun 3;178(7):657–68. doi: 10.1001/jamapediatrics.2024.1484 (PMC11148786; doi:10.1001/jamapediatrics.2024.1484)
Supplement: Supplement 3. — Data sharing statement [file jamapediatr-e241484-s003.pdf]

Data Sharing Statement

Cheung. Immediate vs Gradual Brace Weaning Protocols in Adolescent Idiopathic Scoliosis. *JAMA Pediatr*. Published June 03, 2024. doi:10.1001/jamapediatrics.2024.1484

Data
Data available: Yes
Data types: Other (please specify)
Additional Information: Deidentified data without patient's sensitive and confidential information is available upon reasonable request to corresponding author.
How to access data: cheungjp@hku.hk
When available: With publication

Supporting Documents
Document types: None

Additional Information
Who can access the data: Researchers whose proposed use of the data has been approved
Types of analyses: meta-analyses
Mechanisms of data availability: With a signed data access agreement
Any additional restrictions: Proposed use of dataset cannot violate patient's confidentiality and privacy at any time.
